# Portable Wideband Microwave Imaging System for Intracranial Hemorrhage Detection Using Improved Back-projection Algorithm with Model of Effective Head Permittivity

**DOI:** 10.1038/srep20459

**Published:** 2016-02-04

**Authors:** Ahmed Toaha Mobashsher, A. Mahmoud, A. M. Abbosh

**Affiliations:** 1 School of ITEE, The University of Queensland, St Lucia, 4072, Brisbane, Australia; 2 School of Medicine, Griffith University, Gold Coast, 4215, Australia

## Abstract

Intracranial hemorrhage is a medical emergency that requires rapid detection and medication to restrict any brain damage to minimal. Here, an effective wideband microwave head imaging system for on-the-spot detection of intracranial hemorrhage is presented. The operation of the system relies on the dielectric contrast between healthy brain tissues and a hemorrhage that causes a strong microwave scattering. The system uses a compact sensing antenna, which has an ultra-wideband operation with directional radiation, and a portable, compact microwave transceiver for signal transmission and data acquisition. The collected data is processed to create a clear image of the brain using an improved back projection algorithm, which is based on a novel effective head permittivity model. The system is verified in realistic simulation and experimental environments using anatomically and electrically realistic human head phantoms. Quantitative and qualitative comparisons between the images from the proposed and existing algorithms demonstrate significant improvements in detection and localization accuracy. The radiation and thermal safety of the system are examined and verified. Initial human tests are conducted on healthy subjects with different head sizes. The reconstructed images are statistically analyzed and absence of false positive results indicate the efficacy of the proposed system in future preclinical trials.

Intracranial hemorrhage (ICH), which includes intra-parenchymal, subarachnoid, subdural and extradural hemorrhage, refers to the accumulation of blood inside human head. It happens when a blood vessel inside the intracranial chamber bursts or is abruptly torn as a result of hypertensive damage, physical trauma, and other venous infections and malfunctions. As the regular blood supply to the brain is interrupted, brain cells start dying while at the same time blood swelling elevates the intracranial pressure which compresses the adjacent tissues^1^. Since the ICH (traumatic and non-traumatic) can lead to brain deformation and a huge number of death (mortality rate of 30–50%) and disability (74% survivors) worldwide^2^,^3^,^4^, it is a crucial public health issue. On-the-spot accurate detection by means of head imaging is the governing factor of the timely medication to ensure complete recovery of the injured patient^4^,^5^.

The conventional medical imaging tools like MRI and CT scans are able to reliably diagnose ICH^7^,^8^,^9^. However, the static and bulky structures or ionization radiation of those tools limit their capability for rapid diagnosis at the incident location. Moreover, their high costs (in Australia, for example, more than one thousand dollars per scan) limit their use as monitoring tools and their availability at rural medical clinics. According to World Health Organization (WHO), about three-quarter of the world population does not have access to reliable and affordable medical imaging systems^10^. Hence, a portable and low-cost imaging system is widely demanded for the detection of ICH. Several medical imaging modalities, like electrical impedance tomography (EIT)^11^, magnetic induction tomography (MIT)^12^, magnetic induction spectroscopy (MIS)^13^, or phase shift spectroscopy of magnetic induction (PSSMI)^14^ are recently explored by the researchers. However, these technologies are either invasive or cannot detect ICH in a realistic environment; hence their practical demonstration is scarce.

Microwave based head investigation has recently attracted a huge attention as a non-invasive alternative or complementary non-ionizing diagnostic tool for various biomedical applications^15^,^16^,^17^,^18^,^19^,^20^,^21^. This work reports a portable microwave head imaging system, which is simple to employ and consists of an imaging platform, a compactly designed ultra-wideband (UWB) antenna^22^, a microwave transceiver and a data storing and processing unit. The presented head imaging system utilizes radar based back projection algorithm based on effective permittivity model. One of the prime disadvantages of existing radar based algorithms is the presumption of a constant value for the effective permittivity of the imaged body in order to map the head for any significant scatterer^21^,^23^,^24^,^25^. However, using a constant number for the heterogeneous head that has various tissues leads to significant imaging errors that may lead to incorrect detection and localization^26^,^27^. To address this problem, several researchers have utilized the estimation of direct time of flight of the signal through the imaged body^28^ or time-domain inverse scattering technique that estimates the spatially averaged permittivity^29^. However, in a multipath environment such as the heterogeneous human head, the penetrating signal faces different tissues with wide range of properties and thicknesses. In this work, a novel location-dependent point-of-entry based effective head permittivity model derived using the numerical analysis of a healthy human head is proposed. An improved delay-and-sum (DAS) algorithm relying on the derived permittivity model is then employed for reconstructing an image of the head interior with enhanced detection accuracy. The system and algorithm are validated in realistic simulation and experimental environment by using anatomically realistic three-dimensional (3D) printed human head phantom. The radiation and thermal safety of the system are also verified. Human trials on healthy volunteers are then conducted to determinate the type, statistics and thresholds of obtained images as a necessary step for future preclinical trials.

## Results

### Head imaging system

**Figure 1:**
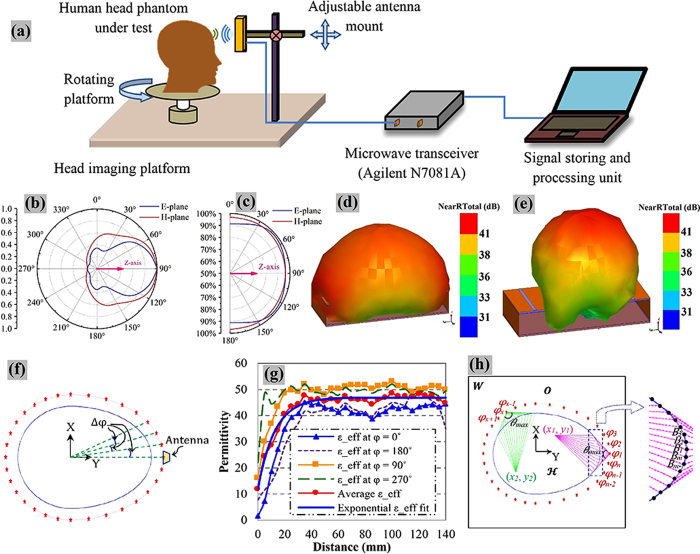
(**a**) Schematic representation of the wideband microwave head imaging system. Time-domain analysis of the antenna element: (**b**) near-field radiation patterns, and (**c**) fidelity factor patterns in both E-and H-planes. An FBR of 6.25:1 is found for the antenna in near-field, which is relatively high considering the electrically small size of the antenna^30^ and proves the unidirectional feature of the antenna in near-field. The antenna exhibits 80˚ and 100˚ half power beamwidth respectively in E and H-planes assuring proper head scanning capability of the antenna. High level of fidelity (more than 95%) is found along the broadside direction (Z-axis) which ensures low level of distortion in the transmitted pulse and resulting in low distorted received pulse. Three-dimensional frequency-domain near field radiation patterns at (**d**) 0. 9 GHz and (**e**) 2.3 GHz. The main radiation beam exists along the +ve Z-axis at 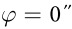 and 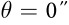. (**f**) Illustration of Equiangular and equidistant scanning system. Readings that are taken from the same distance and equal angular position faces similar reflection from the skin-air interface; hence, it is easier to cancel the strong background reflection in order to obtain the scattering signal from the actual target. (**g**) The effective permittivity data attained from realistic human head model simulations by calculating the delay time of the signal arrival. The effective permittivity values of the head sides (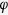 = 90˚ and 270˚) are slightly higher than those of the front and back (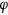 = 0˚ and 180˚) due to the presence of the heterogeneously thick high permittivity muscle (55–52.6 across the band 0.75–2.55 GHz) layer along the sides of the head. (**h**) Path selection of algorithm’s procedure from a particular antenna position to an arbitrary point inside the head.

The architecture of the experimental head imaging system is illustrated in Fig. 1(a). The system primarily consists of a compact antenna to transmit and receive wideband signals, a microwave transceiver for signal generation and data acquisition and a personal computer for signal processing and image formation. A head imaging platform is designed to evaluate the system’s performance on detecting ICH using a realistic head phantom. During image, the head phantom is placed at the rotatable platform and scanned using the wideband antenna, which is mounted on an adjustable antenna holder.

### Data acquisition system

The data acquisition system includes a compact microwave transceiver and an antenna. An Agilent N7081A microwave transceiver is utilized for generating the transmitted microwave signals towards the imaged head via the antenna and collecting backscattered signals with a maximum dynamic range of 80 dB. The utilized wideband antenna^22^ has 109% fractional bandwidth covering the band 0.75–2.55 GHz, which provides a reasonable compromise between the required signal penetration inside the head and acceptable image resolution. The antenna is compact and has low profile (0.26 × 0.11 × 0.05 *λ*_*0*_^*3*^, *λ*_*0*_ = wavelength at 0.75 GHz) with 3.5 dBi gain and 9 dB front-to-back-ratio (FBR) along the boresight direction. Considering the high (around 45) average dielectric constant for the human head over the used band, the far-field region originates approximately from 30 *mm* (2.55 GHz) to 95 *mm* (0.75 GHz) away from the point of penetration^31^. Therefore, near field characterization is equally important for the intended head imaging application. The analysis is performed using electromagnetic simulation tools considering +ve Z-axis as the desired direction of antenna’s radiation. The results shown in Fig. 1(b–e) elucidate that, in near field, the antenna emits directionally with low distortion. Analysing all the near and far-field radiation patterns, it can be concluded that the unidirectional radiation of the antenna (phase center) starts from the center point of top layer over the operating band, enabling to scan a representative 2D layer^32^ of the human head that is located at the centerline of the antenna.

### Signal processing and imaging algorithm

As depicted in Fig. 1(f), the data acquisition system utilizes an equiangular and equidistant scanning approach. The transceiver sends, via the antenna, a frequency-domain signal with *M* = *284* samples covering the band 0.75–2.55 GHz. The transceiver measures via the same antenna (monostatic approach) the complex frequency–domain reflection coefficient, 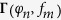 where *m* = 1, 2, ….., *M*. Here, *n* represents the different angular positions of the antenna, which extends from 1 to *N*, while the adjacent antenna’s positions have angular differences of 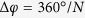. The inverse discrete Fourier transformation is performed to convert the reflection coefficient, 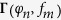 from frequency domain to time domain for each source antenna.


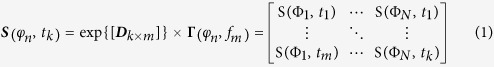



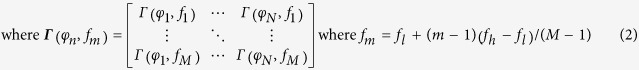



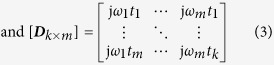


where 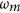 is the angular velocity at the individual frequency points; *c* is the speed of light in free space; *k* represents the equidistant points starting from the antenna’s phase center to the distance where the amplitude of time-domain signal is close to zero. Eqn. (1) considers the positive distances from the point of excitation located in free space.

The transmitted signals are confronted by substantial amount of reflections from the air-skin interface. To extract the useful scattered signals from the internal tissues of the head, it is assumed that as the antennas are positioned in close proximity to each other, the adjacent positioned equidistant antennas face similar reflections from the air-skin interface. Hence, the scattered signals are computed by


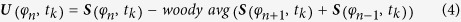



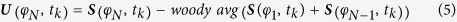


The adjacent average subtraction takes the advantages of both adjacent^33^ and average^34^ subtractions. In this case, Woody average (average with first alignment of individual signal) is utilized which is also proven to be effective for biomedical and imaging applications^35^.

The variation of the antenna gain over the wide bandwidth is compensated by normalizing the amplitude of the scattered signals by


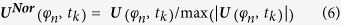


The imaging area of 300 × 300 *mm*^*2*^ is segmented into P×Q cells, having fine resolutions of 0.5 × 0.5 *mm*^*2*^, which are defined by their center points at (*x*_*p*_, *y*_*q*_). The head outline is defined by a boundary vector, 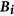 where *i* = 1, 2, …., *I*, located at the skin-air interface in the Euclidean space. The propagation path from the antenna to a hypothetical point inside the head consists of: *1)* a route in free space 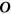 from the emitting source to the skin layer and *2)* path inside the head 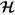 extending from the skin layer to the investigated point. According to Fermat’s principle, the wave propagates along the shortest possible electrical path ***D*** and thus the propagation time index 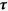 can be calculated as,


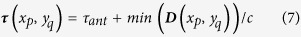


where 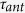 is the antenna delay, defined as the time required to emit electromagnetic radiation from the antenna exterior from the inception of the signal at the feeding port; ***D*** is defined by,


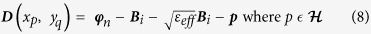


Here 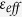 is the effective head permittivity which is a constant number in the previously utilized algorithms^21^,^23^,^24^,^25^. A model for the effective dielectric constant as a function of signal’s point of entry and imaged location within the head is utilized in the proposed algorithm.

When a signal enters into head, it has to interact with an irregularly thin high permittivity skin (50–44 across the band 0.75–2.55 GHz) layer and then penetrates through thick fat and skull layers having low permittivity. Afterwards, the signal goes through very high permittivity CSF (69–66.2 across the band 0.75–2.55 GHz) and Dura (44–41.9 across the band 0.75–2.55 GHz) tissues, and gradually passes through grey and white matters. The models found in the literature reporting the binary liquid mixture law conclude that the value of the effective permittivity of a mixture of two materials inclines toward the liquid with higher volume. If the volume of higher permittivity liquid is more than the volume of lower one, the resultant permittivity value falls near to the higher permittivity value^36^,^37^. In order to extend this phenomenon to the heterogeneous human head, a realistic simulation model with a MRI-derived healthy human head is analysed in CST Microwave Studio environment over the band 0.75–2.55 GHz. Fig. 1(g) exhibits the effective permittivity graphs of the healthy human head from various 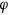 angles. The effective permittivity is calculated by analysing the differential time of signal arrival from the point of entry. It is seen that the effective permittivity starts from a small value at the point of entry due to the thick low permittivity layers, gradually increases as the signal interacts with thick permittivity values, and subsequently reaches a maximum effective permittivity value and remains around that value for the rest of the propagation distance. The effective permittivity of an arbitrary point inside the head is different for various points of entry of the signal and related to the distance of that arbitrary point from the skin. To have a unified propagation model, the values are averaged and the result is expressed using a distance based function for a particular point of entry of the head,


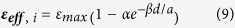


Here 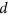 is the distance of the investigation point, *a* is the normal distance from the signal entry point to the center of the head, 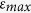 is the maximum permittivity, and 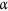 and 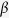 are constants. For the band of 0.75–2.55 GHz, the effective permittivity values from the realistic simulation environment suggests values of 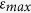 = 46.8, 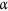 = 0.75 and 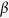 = 6.4.

In mono-static radar approach, the transmitted signal travels to and scattered from the arbitrary point; thus, the required total time from the antenna is twice of the propagation time


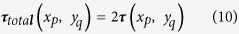


To reduce the computation complexity, it is realized that the points located on the skin (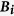) receive a direct wave from the antennas through free space and that consequently results in a minimum propagation path. Hence, the line of sight points of each antenna (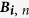) are traced out by calculating the tangent points of the 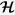 area and the points between them (Fig. 1(h)). The tangent points can be calculated by finding the maximum angle, θ_*max*_ centered at the antenna location. Thus, (6) can be written as,


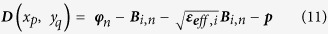


Utilizing (5), (8) and (9), the total time, 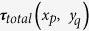 is calculated for every (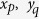) points of 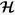 region.

The delay-and-sum back-projection algorithm is employed for the image reconstruction. According to this algorithm, it is assumed that the normalized scattered signals, 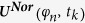 are originated from a given point 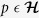 and a coherent summation of the signals originating from surrounding antenna is computed.


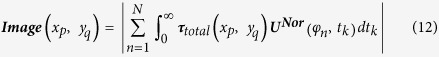


If this hypothetical scattering point is correct, the coherent summation of all the antenna responses results in a large value. Wrong assumption gives a small value; this contribution can be considered as noise. A continuous colour image is produced by plotting the computed 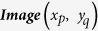 function for each point on the segmented 2D cross-sectional scanned plan of the head phantom. High intensity (usually normalized, thus highest intensity is 1) of colours indicates positions of significant scatterers (hemorrhage) inside the head.

### Performance evaluation in simulation environment

**Figure 2:**
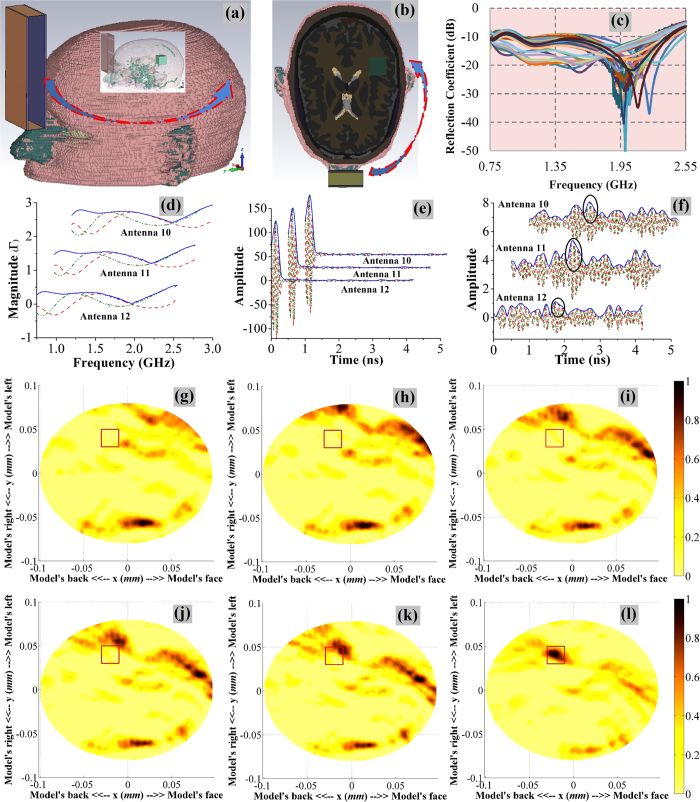
(**a**) Perspective view of the 3D head model with an inset showing the target location, and (**b**) top view of the simulation environment. The arrows point toward the direction of antenna rotation around the 3D head model. (**c**) The raw data received by the antenna in different positions. (**d–f**) Various signals from closest antennas 10, 11 and 12 in the processing pipeline: (**d**) the reflection coefficient data, 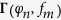, (**e**) the time domain converted data, 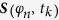 and (**f**) the refined scattered signals, 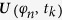 after the adjacent average subtractions. Here the red, green and blue curves respectively represents the real, imaginary and absolute values of different signals. The suspected positions of the target is circled in 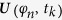 signal. (**g–l**) The reconstructed images of the 3D human head model after simulating the head imaging system in numerical environment including all the frequency dispersive electrical properties of the human head with a haemorrhagic target inside. The images are reconstructed using the existing algorithm with fixed effective permittivity of (**g**) 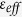 = 30, (**h**) 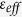 = 35, (**i**) 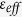 = 40, (**j**) 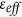 = 45, (**k**) 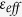 = 50 and (**l**) proposed model of effective permittivity. The red rectangle shows the actual position of the head injury.

In order to validate the head imaging system along with the utilized signal and image processing algorithm, a set of full-wave simulations are performed in the presence of a 3D human head model. Fig. 2(a,b) depict the realistic 3D head model and the direction of scanning. A signal-to-noise ratio (SNR) of 30 dB, which is usual in real-life environment, is selected for the simulation. Fig. 2(c) shows the reflection coefficient results of the antenna in different positions. It is seen that the antenna reacts differently according to the material (tissue type and thickness) placed in front of it.

The received signals are translated into images of the head interior using the aforementioned approach. The treated signals in different stages of the data processing pipeline is also demonstrated in Fig. 2(d–f). Fig. 2(g–l) depict a comparative illustration of the reconstructed images using both the existing^21^,^23^,^24^,^25^, and the proposed algorithm. It is noted that with existing algorithm, the reconstructed images significantly vary with the assumed effective permittivity values. However, the proposed algorithm is more accurate in reconstructing the target location inside the head interior.

### Analysis of the simulated results

The efficacy of the compact head imaging system and proposed algorithm is analysed comparing the image quality with the images obtained from the existing fixed average permittivity based imaging algorithm^21^,^23^,^24^,^25^. Several quantitative matrices are investigated as defined in the methods section.

Considering images from simulation environment, qualitative and quantitative matrix analyses of pre-known hemorrhagic location and size reveal that the existing algorithm using fixed 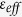 fails to identify the location of the target. While a sense of target localization is noted in case of 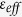 = 50, another substantial artifact exists in the frontal area which results low signal to maximum clutter and contrast ratios with wrong detection. Details of the quantitative matrices are listed in Table 1. On the other hand, the proposed algorithm distinctly identifies the position of hemorrhagic target. The resultant image of Fig. 2(l) presents higher amplitude ratio of 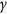 = 1.39 and contrast of 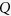 = 5.83. The image demonstrates very low deviation of 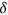 = 0.71 *mm* from the center of hemorrhagic position to the highest amplitude. Based on the quantitative matrices, it is noted that the proposed algorithm is more accurate in reconstructing the target location inside the head interior.

### Map of reconstruction accuracy

**Figure 3:**
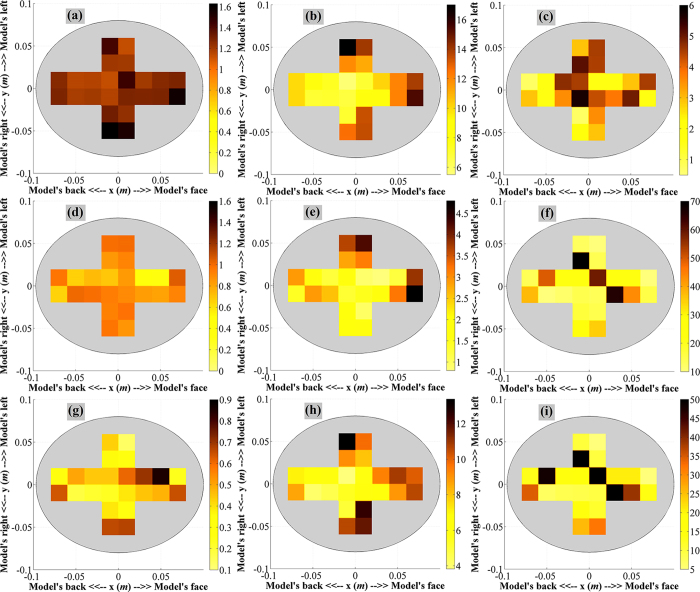
(**a–c**) The map different quantitative matrices demonstrating the reconstruction accuracy of the realistic human head model using the introduced back-projection algorithm. (**a**) The map of signal to maximum clutter function, 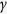. (**b**) The map of average signal to clutter ratio function, Q. (**c**) the distance between the true central location of the target, 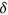. (**d–f**) The map different quantitative matrices demonstrating the reconstruction accuracy of the realistic human head model using the existing back-projection algorithm relying on constant effective head permittivity of 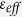 = 45. (d) The map of signal to maximum clutter function, 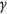. (**e**) The map of average signal to clutter ratio function, Q. (**f**) the distance between the true central location of the target, 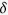. (**g–i**) The differential map of the head model’s cross section demonstrating the improvement of image reconstruction when the proposed modified back-projection algorithm is compared to the existing one. (**g**) The map of differential signal to maximum clutter ratios defined as, 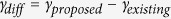. (**h**) The differential map of average signal to clutter ratio functions defined as, 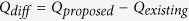. (**i**) Difference map of the distances between true central location of the targets defined as, 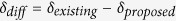.

To systematically validate the potential of the system, a map of its image reconstruction accuracy is generated through systematically choosing 24 positions along the horizontal X and Y axes of the imaged layer. The details of the ICH target positions are described in Supplementary Fig. S2. The reconstructed images using the proposed modified back-projection and existing algorithms of every position are illustrated accordingly in Supplementary Figs. S3–S26 (b) and Supplementary Figs. S3–S26 (c). Fig. 3(a–c) demonstrate the maps of the quantitative analysis of the reconstructed images using the proposed algorithm. It is noted that for every location, the signal to maximum clutter function, 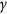 is more than 1 indicating the ability of the algorithm to detect the ICH targets effectively. However, the detection sensitivity is different from one location to another. Maps of similar quantitative investigations from the existing algorithm relying on a fixed head permittivity (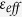 = 45) are also shown in Fig. 3(d–f). Finally, quantitative comparisons between the existing and proposed algorithms are presented in Fig. 3(g–i). This differential map demonstrates significant quantitative improvements in the detection capability of the proposed algorithm.

### Experimental validation of the head imaging system

After the performance evaluation in the simulation environment, the head imaging system is tested experimentally. A realistic human head phantom is constructed for the validation purpose. The phantom is fabricated by emulating the electrical properties of various tissues of human head, such as gray matter, white matter, Dura, cerebellum, CSF, and so on. The exterior structure (skin, fat, muscular parts and skull) and the interior moulds of the head phantom are built in 3D printer^38^. The exterior is utilized to hold the internal brain parts which are fabricated with tissue emulating materials. Additionally, skin emulating viscous liquid is also utilized to include the effects of the skin^39^.

The experimental flow chart of the data collection and storing process is illustrated in Supplementary Fig. S1. Following the sequences, the scanned reflection coefficient signals, from which the backscattered signals are extracted, are stored in a matrix 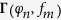 for post processing.

**Figure 4:**
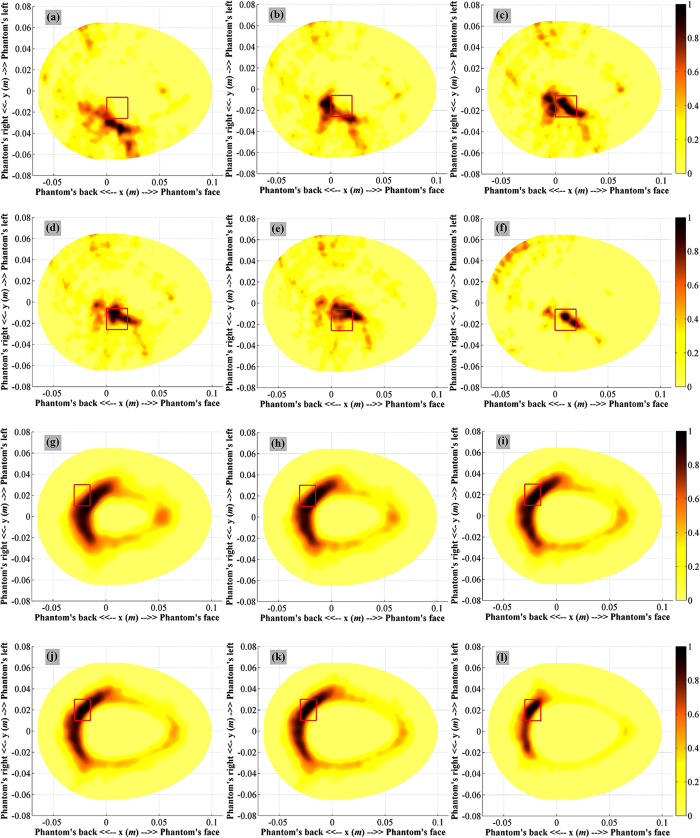
Head imaging results of target-1 (2 × 2 × 0.5 cm^3^) ICH target in deep location using different algorithms: fixed average permittivity based algorithm with **(a)** 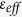 = 30, **(b)** 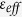 = 35, **(c)** 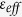 = 40, **(d)** 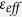 = 45, **(e)** 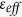 = 50 and **(f)** algorithm relying on model of variable effective permittivity. Head imaging results of a small (1.5 × 2 × 0.5 cm^3^) ICH target in mid location using different algorithms: fixed effective permittivity based algorithm with (**g**) 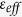 = 30, (**h**) 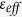 = 35, (**i**) 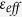 = 40, (**j**) 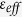 = 45, (**k**) 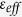 = 50, and (**l**) algorithm relying on proposed model for variable effective permittivity.

Validation of the system and algorithm of both target-1 and -2 (ICH) in different locations are performed. The collected data is utilized to reconstruct images of the head interior. The resultant images from the proposed algorithm is then compared with the images received by using the traditional method with fixed effective permittivity based algorithm^21^,^23^,^24^,^25^. A range of fixed average permittivity from 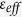 = 30–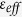 = 50 are assumed for the whole head to observe the variations of the resultant images. It is seen from Fig. 4 that the proposed algorithm provides better reconstructed image for both deep and shallow targets.

### Analysis of experimental results

The results confirm the effectiveness of the proposed model for the effective permittivity in head imaging. In case of target-1, the fixed effective permittivity based algorithm illustrates several artifacts, which can be corrected by utilizing the proposed algorithm. As seen in Table 1, the maximum intensity ratio, 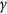 and average intensity ratio 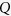 of the proposed algorithm is higher than the reported algorithm of^21^,^23^. The calculated accuracy indicator, 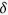 considers the maximum intensity point closer to the center of the target region which indicates higher accuracy of the target recognition. Although in case of 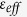 = 45, the 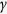 value is comparable to that of the proposed algorithm, the other values are relatively low, demonstrating the image quality enhancement using proposed algorithm. For the small target, the proposed algorithm results in improved values of the calculated 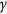, 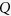 and 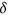 parameters. It indicates that as the target goes closer to the skin the scattering signals from the target becomes stronger and hence, the algorithm is able to reconstruct the target with increased accuracy in comparison to the previously developed algorithms.

### Effect of Noise

**Figure 5:**
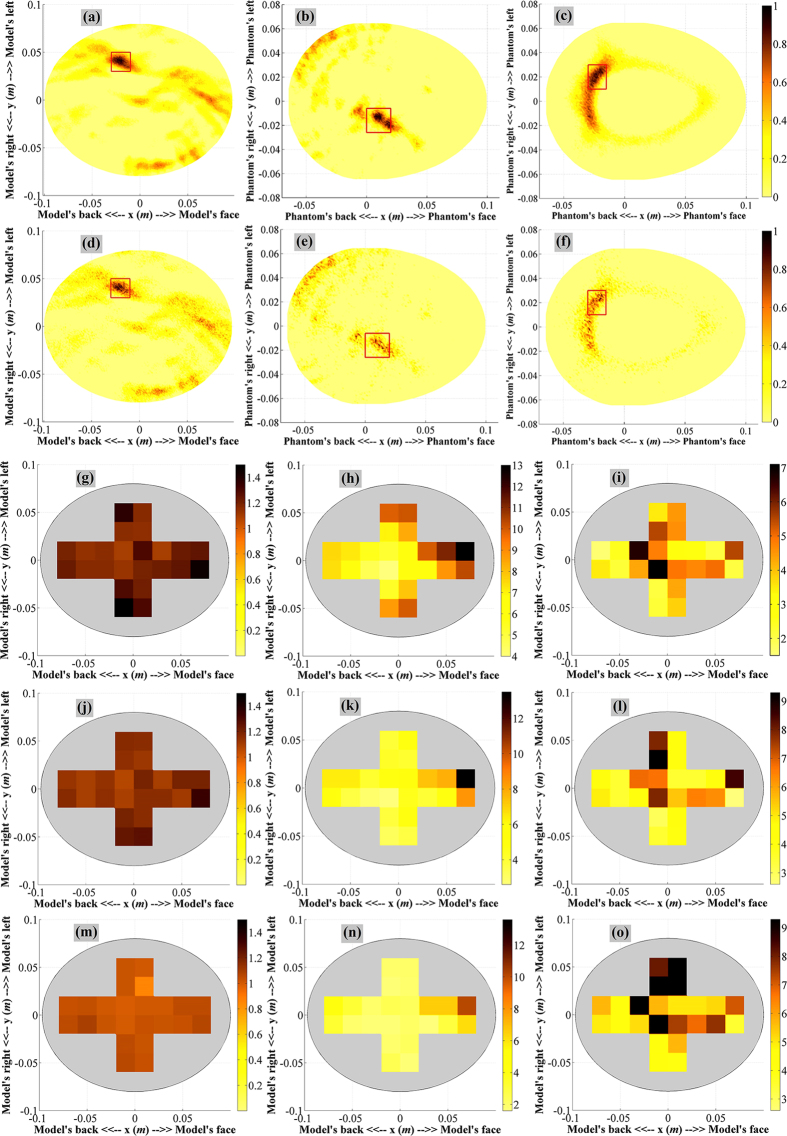
(**a–c**) The simulated (**a**) and measured (**b,c**) reconstructed images at SNR = 20 dB. (**d–f**) The simulated (**d**) and measured (**e,f**) reconstructed images at SNR = 10 dB. The maps of the quantitative investigations, namely 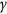, 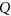 and 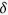 accordingly, demonstrating the reconstruction accuracy at (**g–i**) SNR = 20 dB. (**j–l**) SNR = 10 dB, and (**m–o**) SNR = 5 dB. The original reconstructed images are depicted in Supplementary Figs S3–26 (**d–f**).

The effects of noise on the proposed head imaging system and its reconstructed images are also investigated. The SNR values of the simulated and measured images are gradually decreased from 30 dB–10 dB. Fig. 5(a–c) show the simulation and measured results when the SNR = 20 dB, whereas Fig. 5(d–f) report the reconstruction at SNR = 10 dB. The quantitative results of the reconstructed images are listed in Table 1. Nevertheless, maps of the quantitative analyzes of the image reconstruction are also plotted in Fig. 5(g–o) for the gradual SNR reduction, while the differential maps are presented in Supplementary Fig. S27. As expected, a substantial diminution of image quality is noted for a significant reduction in the SNR.

### Safety considerations of the system

**Figure 6:**
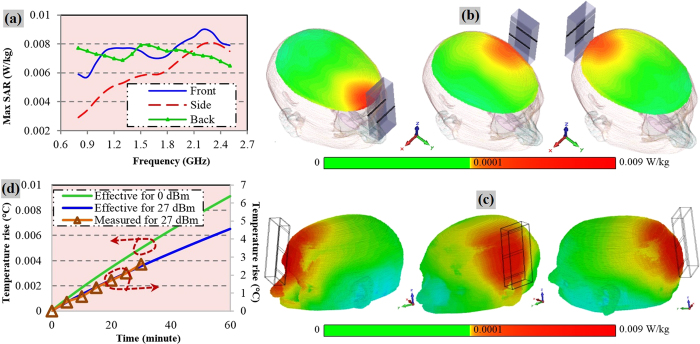
Clockwise. (**a**) The calculated maximum SAR over the functional bandwidth for antennas operating from various directions. The maximum SAR values induced by the antenna of the system are less than 0.01 W/kg for the whole band of operation. (**b**) Cross-sectional view of the realistic human head model while the antenna is operating at 0.8 GHz from various directions. (**c**) Perspective view of the head model while the system is operating at 2.4 GHz. (**d**) Simulated and measured effective skin surface temperature rise due to the electromagnetic emission from the antenna. From the governing eqns. (16–18), it is noted that the skin temperature is highly sensitive to the operating frequencies and the higher frequencies increase skin temperature much quickly than the lower ones. This is due to the increase of skin’s conductivity at higher frequencies that reduces the skin depth (penetration depth) of the skin tissues resulting in the accumulation of thermal energy due to electromagnetic losses around the skin surface.

The radiation safety of the patient while applying the presented detection and monitoring device is also investigated. Since the system works in mono-static manner, only one antenna is active at any particular time even if an array of antennas is used for fast data acquisition. Each of the antennas transmits 0 dBm or 1 mW power toward the head and receives the backscattered signals. The specific absorption rate (SAR) of the head is calculated by using CST simulation tool. An MRI-scan based in-house built human head phantom is utilized for this purpose where the frequency dispersive electrical properties of the actual tissues are employed^40^. The head model and the SAR results are presented in Fig. 6(a,b). It is observed that the SAR values are well below the IEEE safety limit of 2 W/kg^41^,^42^. The mid-cross section illustration of the head model while the system operates at 0.8 GHz (Fig. 6(c)) reveals the gradual dissipation of the emitted signal. Again, Fig. 6(d) presents the system at 2.4 GHz and it is noted that most of the power loss is located at the skin layer of the human head especially at the nearest point from the middle of the antenna.

Since the system might be active over a long time for continuous monitoring of the patient, the thermal effect of the electromagnetic signals is also a matter of interest along with SAR verification. The effect on skin is particularly important as skin absorbs most of the emitted power and heating from the system might cause skin burns or discomfort to the patient. With the transmitted power of the antennas, at 15 *mm* distance, the incident power density on the skin is calculated as 1.24 W/m^2^ or 0.124 mW/cm^2^, which is less than the radiation safety limit^43^,^44^ of 1 mW/cm^2^. Since the scanning approach of head imaging system operates by sweeping from lower frequencies to higher ones (0.75–2.55 GHz) for an individual reading, all frequencies do not operate at the same specific moment. The effective rises in the temperature of skin at particular times are calculated and presented in Fig. 6(d). However, as the temperature rise is comparatively low for the used thermal camera, the measurement is performed by using higher transmission power of 27 dBm (=0.5 W) for the sake of validation. It is seen that the measured skin temperature rise for 27 dBm matches the calculated results, which validates the previous calculated temperature rise for 0 dBm, indicating the safety of the head imaging system.

### Pilot human tests of the system prototype

**Figure 7:**
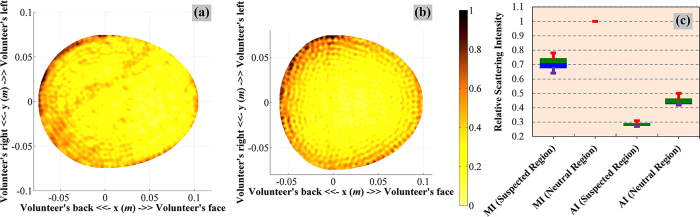
(**a,b**) Examples of the reconstructed images of healthy volunteers using a prototype of the head imaging system. (**c**) The statistics of the maximum and average scattering intensities of the neutral and suspected regions of the volunteers. The green and blue boxes represent the 75th and 25th percentiles respectively and the transitional line between them presents the median. The red and violet lines accordingly states the maximum and minimum magnitudes. MI = Maximum intensity, AI = Average intensity. The MI of neutral region in all regions is one, as the images are normalized with respect to the highest intensity and it falls in the neutral region.

A prototype of the head imaging system is utilized to analyse the images that can be obtained from healthy volunteers with different head shapes and sizes. Samples of the reconstructed images of the volunteers depicted in Fig. 7(a,b) illustrates that in any healthy volunteer’s head the most significant scatterers are located along the skin layer. This scenario demonstrates that in healthy volunteers the skin exhibits the strongest scattering compared to the other brain parts. The interior of the head does not pose any substantial scattering; hence, the images are free from false positive responses. Since ICH does not occur in the skin or skull region^3^,^4^,^45^,^46^, the skin and skull tissues (which are about 10 *mm* thick) can be considered as a neutral region, whereas the rest head interior as the suspected region. To that end, the images of healthy volunteers are statistically analysed (Fig. 7(c)). It is found that the median of maximum and average intensities of the neutral region are around 1.4 and 1.5 times higher than those of the suspected region.

Although all images are normalized with respect to the maximum intensity of that individual image, extended observations indicate that while the un-normalized maximum magnitude due to hemorrhagic target in the simulated (Fig. 2) and experimented (Fig. 4) phantoms with hemorrhagic is in the scale of 10, the experimental images (Fig. 7(a,b)) from the human subjects exhibit highest intensity of around 1. Thus, it is anticipated that in the presence of ICH, the scattering from the neutral region is going to be negligible compared to that of bleeding and there might be a possibility of imaging threshold. However, further investigation of healthy and unhealthy subjects is required to finalize the conception of the imaging threshold.

## Discussions

A simple and effective head imaging system operating from 0.75–2.55 GHz is introduced in this paper. A data acquisition system, including a compact microwave transceiver and a low-profile UWB antenna, is utilized to collect the backscattered signals. The near-field characteristics of the antenna are analysed to verify suitability for system application and the antenna is found to be unidirectional with low distortion radiation performance compared to the previously reported compact antennas^6^. Furthermore, the point–of-entry dependent permittivity based delay-and-sum algorithm is introduced in this manuscript which has demonstrated its potential by providing enhanced image quality and accuracy compared to the previously developed fixed effective permittivity based algorithm. The efficacy of the head imaging system is firstly verified on a 3D human head model with a hemorrhagic target in simulation environment. It is noted that the target’s response (Fig. 2(d–f)) is masked in the background and the modified processing algorithm is able to uncover the scattered signal from the target. Reconstructed images from simulation data reveals that the fixed permittivity based algorithm shows a lot of artifacts and fails to detect the actual position of the hemorrhage Figs. 2(g–k), while the proposed algorithm is able to detect its actual location Fig. 2(i).

In the first simulated model (Fig. 2), the ICH hemorrhagic target is positioned along the edge of gray matter tissue. It is found that the system can detect ICH with low contrast which can be explained as follows. The wave impedance that combines both dielectric properties (permittivity (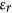) and conductivity (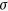)) for the operating frequencies can be calculated by, 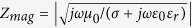, where 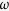 is the angular velocity, 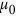 is absolute permeability and 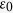 is absolute permittivity. 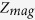 of blood ranges from 44.8–49.9 Ω over the same operating band; whereas for gray matter, it is 48.9–52.5 Ω. This gives a wave impedance contrast of 9.2–5.2% between the hemorrhagic target and the background (gray matter) across the band from 0.75–2.55 GHz. However, deep targets with white matter background have contrast ratio of around 27.5–23.6% over the band. This higher contrast assists in ICH detection. However, in deep locations, owing to the increase of penetration losses, the average signal to clutter ratio function, Q decreases with an increase of localization error, 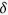. This effect can be realized from the maps of the image reconstruction accuracy Fig. 3(a–c).

The head imaging system is practically validated on a 3D printed human head phantom having frequency dispersive electrical properties by using the prototyped data acquisition system (SNR = 30 dB) and the controlling software. Signals are measured for both target-1 (2 × 2 × 0.5 cm^3^) and target-2 (1.5 × 2 × 0.5 cm^3^) hemorrhagic targets in different locations with high and low contrast ratios respectively. The measured signals are post processed and images are reconstructed using both algorithms. The quantitative analysis discloses that the proposed algorithm demonstrates better image quality with clear and easily distinguishable bleeding location. However, due to the penetration losses mentioned before, the shallowly located targets are easier to detect (Table 1). These resultant matrices of the proposed algorithm are comparatively higher with much accurate detection capacity than those of the existing algorithm and even the reported values^21^,^23^.

A comparison between simulation and measurement with similar setup can be performed. In this case, reconstructed images presented in Fig. 4(f) and Fig. S14(b) (in the supplementary file) are considered, where ICH target is located at the centre of the frontal right quarter of the head with 2 × 2 cm^2^ cross section. Quantitative analyses reveal that with SNR=30 dB, the reconstructed image from simulation attains better detection and localization than that of measurement. For simulation, the values of the matrices are *γ* = 1.3, *Q* = 10 and *δ* = 0.7 *mm*, while from measurement the values are *γ* = 1.17, *Q* = 9.9 and *δ* = 3.54 *mm* respectively. The similarity in values illustrates the efficiency of image reconstruction. However, the realistic head phantom in the measurements is smaller than the simulated head model, which is acceptable as different patients will have different head shapes and sizes. It is realized that with a bigger head model, the detection and localization ICH target with same contrast and position will tend to degrade as concluded from discussions pertaining Fig. 3(a–c).

The gradual SNR reduction from 30 dB–5 dB as a result of increased noise demonstrates (Fig. 5) the gradual reduction in image quality, yet the shallower ICH targets show higher quantitative matrices than the deeper ones. Nevertheless, the differential quantitative studies from one SNR to another indicate that shallow ICH targets are also more sensitive to change in SNR than the deep ones. Quantitative investigations of both simulations and measurements reveal that if SNR < 10 dB, the system heavily sacrifices the image quality and mostly unable to detect or falsely detect ICH targets. However, real-life environment usually provide SNR of more than 10 dB.

The system has the potential to be operated for continuous quasi- real time monitoring purposes of head injured patients. For this reason, the safety issues of the head imaging system including the radiation and temperature safety are checked to avoid any operational danger. The SAR values of the system are found to be much lower than the IEEE safety limit ensuring the electromagnetic safety of the system. The temperature rise at the skin layer is also computed for 60 minutes of realistic frequency swiping operation over the frequency band. It is observed from Fig. 6(d) that the temperature rise is not significant even after the continuous operation of the head imaging system for 60 minutes. However, it is found that for the utilized level of power, the temperature rise is very small. Temperature rise is also verified for 27 dBm transmission power through measurement, which proves the effectiveness of the calculated estimations.

The sources of the simulated and measured phantoms are different. Hence, although the simulated and measured antennas in free space show the same performance, the experimental results are not exactly the same as the simulation when the antennas are placed in front of the phantoms. However, appropriate reconstruction of the ICH target reflects the effectiveness of measurements.

A system prototype is developed to perform a pilot human test on two healthy volunteers. A significant scattering from the skin is noted from Fig. 7(a,b). This is attributed to the residual scattering response of the skin after the adjacent average subtraction. This residual scattering from the skin can also be seen from the 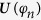 signal of Fig. 2(f). Due to slight variation of skin thickness between adjacent positions, it can be seen that residual skin scattering present in 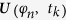. However, they are not dominant for the scenario where there is an ICH target. For this reason, even though the simulated model and the emulated measured phantom have skin response, it is not significant when compared to that of ICH target. But these skin reflections plays vital roles at times when there is no ICH inside the patient, as seen for healthy volunteers. In healthy head, no significant target coherently adds up like ICH target and hence the skin becomes the significant scatterer.

The head imaging system can successfully detect targets of volume 2 × 2 × 2 cm^3^ in simulation and 2 × 2 × 0.5 cm^3^ in one of the measurements. In another measurement, the system is able to detect an even smaller target of 1.5 × 2 × 0.5 cm^3^. At early stages, the suspected ICH patients exhibits ICH targets with a median of 17 cm^3^ 45 or in the scale of centimeters^46^. Therefore, the detectable amount by the system is more than adequate for the early stage ICH detection. Because of the reduced computational complexity, the image reconstruction with proposed algorithm takes 7–10 seconds, which is less than the time required by existing algorithms^21^,^23^. Other reported low-cost and portable imaging technologies (e.g., EIT^11^, MIT^12^, MIS^13^, PSSMI^14^) demonstrate advantages in either simulations or on simple phantoms without much realistic demonstration. Nevertheless, tomography based imaging system requires more data acquisition and long computation time^47^. However, at this point of research, pilot human tests are limited to healthy volunteers. Hence, although the tests illustrate the absence of false positive results, more measurements of healthy and unhealthy subjects are required for a successful preclinical system.

The system is compact and light weight (less than 1 kilograms including the data acquisition system) which can be applied as a portable system. Although data acquisition process takes 3–4 minutes it can be made less than 30 seconds by using an array of antennas. The future works involve implementation of the array of antennas in a compact package. Thus, the future generation of portable head imaging system can consequently be sent for preclinical trials to detect head injuries.

## Methods

### Near-field time-domain radiation pattern analysis

In order to analyse the near-field performance of the antenna, finite-difference time-domain (FDTD) method based electromagnetic simulator, CST Microwave Studio is utilized. Several time-domain near-field probes are placed at 25 *mm* distance from the antenna surface with 10˚ angular difference around the antenna. The antenna is excited with an input pulse that can be expressed as,


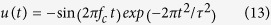


Here, central frequency, *f*_*c*_ = 1.6 GHz and Gaussian pulse width, 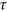 = 1.7 ns are employed to center the pulse spectrum center in the operating band covering 0.75–2.55 GHz in frequency domain^48^. Near-field probes are used to receive the transmitted pulses in both E and H-planes. The received time domain signals are analysed in MATLAB to calculate the near field time-domain radiation and fidelity factor^49^ patterns which exhibits the degree of distortion inherently imposed by the wideband antenna in time domain. The patterns are illustrated in Fig. 1(b,c).

### Near-field frequency-domain radiation pattern analysis

The near-field frequency-domain 3D radiation patterns are calculated by using finite element method (FEM) based simulation software HFSS (ANSOFT). The excitation of the antenna is performed by using a wave port which emulates the utilized SMA connection feeding. The boundary of the near-field radiation calculation is defined as a sphere with 70 *mm* radius, while the antenna is placed in air medium. The radiation patterns are superimposed on the antenna structure to clarify the position and direction of the radiation. These are shown in Fig. 1(d,e).

### 3D electromagnetic scanning in simulation environment

The electromagnetic simulation tool CST Microwave Studio is utilized in this regard. To mimic the realistic scenario in the simulation environment, a 3D human head model is created in MATLAB environment using high-resolution magnetic resonance imaging (MRI) scans of a real patient. The model comprises 128 transverse slices and consists a three-dimensional grid of 256 × 256 × 128 cubical voxel elements while the voxels have dimensions of 1.1 × 1.1 × 1.1 *mm*^*3*^. Using MATLAB, a small amount of hemorrhage (bleeding) of 20 × 20 × 20  *mm*^*3*^ is inserted into the model to emulate the hemorrhagic target scenario. The bleeding is positioned in the intended investigation area. The model is imported in CST Microwave Studio and each tissue types are assigned with respective frequency dispersive electrical properties of actual human tissues^50^. The hemorrhagic voxel elements are defined according to the properties of blood. The antenna element is placed 15 mm away from the 3D human head model and a set to simulations are carried out over the frequency band of 0.75–2.55 GHz using the equiangular and equidistant scanning approach. A parabolic scanning profile is selected by using intersections of the *N* = 32 number of vertices of the parabola in 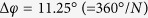 angular differences. The indices (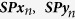) of the parabolic scanning profile can be defined as:


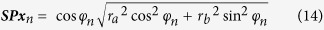



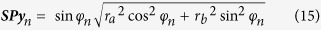


where 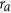 and 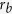 are radii of the parabolic path and 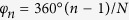, 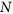 is the total number of scanning positions. Simulation time required for each antenna position is 7–8 minutes using a computer with 3.4 GHz processing speed and 8 GB RAM in a 64-bit operating system. Results from the simulation model are demonstrated in Fig. 2(c).

### Effective permittivity model simulation

In order to get a realistic effective permittivity map of the human head interior, a simulation model is created in CST Microwave studio. The MRI based realistic human head model is imported and assigned with the actual dielectric properties of individual head tissues. The radiating antenna is placed in front of the point of entry of the human head, which is theoretically at the transition of air and skin layer. Electromagnetic field probes are inserted in different distances into the head model and the antenna is excited with the frequency band of 0.75–2.55 GHz and run in time-domain solver. The received signals are analysed and the difference between the times of signal arrival, 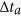 is calculated for every field probe inside the head from the reference entry point field probe. The effective permittivity from the point of entry is calculated as, 
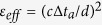
, where 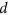 is the distance from the entry point to the particular investigation point and 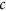 is the speed of light in free space. The acquired data received from the simulation model is presented in Fig. 1(g).

### Image reconstruction

The stored data matrix is imported in MATLAB and processed using the code and reconstructed images are presented in various figures.

### Quantitative Analysis

Firstly, the position of maximum intensity is calculated to check whether it falls inside or outside the actual target position. The signal to maximum clutter function, 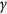 is defined by comparing the maximum intensities of all the points inside the target location and outside it, but within the head area. If the whole head and target regions are, respectively, defined as 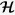 and 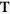,


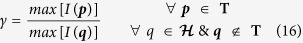


The value of 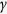 quantifies the presence of any artifact outside the location of the intracranial target. If 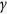
<1, the position of maximum intensity is outside the actual region (incorrect localization) and the cases where 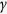
>1 the highest peak is inside the actual target area (correct localization). The value of 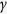 closer to unity indicates how revealing another artifact is compared to the actual target.

Since the reconstructed image plots significant scatterers with high signal intensities, target recognition from the image can be quantified by the average signal to clutter ratio function, Q which is defined as the relative magnitude of the average intensity values of the points inside the actual target region, 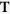 to the rest of the head area,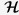.


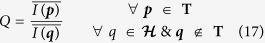


Higher values of 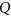 indicates that the ICH can be identified easily from the reconstructed image.

The accuracy of target localization in the resultant image can be described by the parameter, 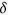 which is defined as the distance between the true central location of the target, 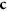 and the position of the estimated maximum intensity on the reconstructed image. This accuracy indicator is denoted as,


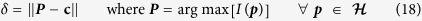


In ideal case, 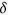 has to be zero, but due to the heterogeneous structure and frequency dispersive properties of the actual head tissues, this value is hard to attain.

### Effect of noise analysis

The investigation of noise effect is performed following ref. 51. Since the wideband imaging system can be viewed as having Gaussian noise distribution, various Gaussian noise figures are added to the received signal. The signals are then analysed through the proposed post-processing algorithm and the resulted images are quantitatively analyzed.

### SAR estimation

The simulations to estimate the SAR level is performed by using CST microwave studio tool. The previously developed 3D human head phantom is imported from MATLAB and the respective tissue types are assigned with their actual measured values^50^. The simulation done by using customized meshing using 20 lines per wavelength with a refinement of lossy metal edges. The antenna is placed in different positions around the head and SAR is then estimated by using template based post-processing action. 10g SAR is calculated by monitoring power loss density and stimulated excitation power.

### Effective temperature rise calculation

The rise of temperature at the skin from the emitted power of an antenna can be calculated by^44^,


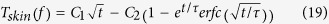


where


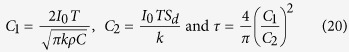


Here, 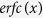 is the complementary error function and coefficient of surface transmission of skin is, 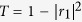, while reflection coefficient of skin/air interface is expressed as, 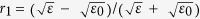. Here complex permittivity of the skin, 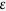 is dependent on the operating frequency, 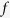 and absolute relative permittivity of air, 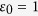. Thermal inertia of skin, 
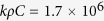
 W^2^s/m^4^°C^2^ and thermal conductivity of skin, 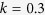 Wm^−1^˚K^−1 ^43,44. The thermal time constant 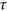 is defined as the time needed for thermal wave to diffuse skin depth (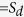) equivalent distance.

The effective temperature rise of the skin at a particular time can be calculated by,


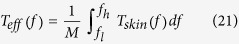


### Temperature measurement

The temperature rise of the skin is measured while an antenna element is placed in front of the 3D printed human head phantom. An infrared thermal imaging camera (Thermo Shot F30S from NEC Avio Infrared Technologies Co. Ltd.) is used to capture the temperature differences at 5 minutes time differences. The maximum temperature rise is measured around the skin region where the antenna is positioned while keeping the measurement time at its minimal so that there is very low chance of error due to environmental cooling of the skin surface. The measurement is performed in an anechoic chamber facility to reduce the external interferences and to increase the radiation safety of the personnel as the measurement involves relatively high input power of 27 dBm. The measured result is presented in Fig. 6(d).

### Head imaging of human subjects

A prototype of the head imaging system is developed in order to test on healthy volunteers. The prototype is equivalent to the system illustrated in Fig. 1(a). The experimental protocols are approved by the Medical Research Ethics Committee (MREC), and Behavioural and Social Sciences Ethical Review Committee (BSSERC) of The University of Queensland for research involving human participants. The experiments on volunteers are performed in accordance with the relevant guidelines and regulations approved by the ethical committees. The participation of the volunteers is maintained in accordance to the approved guidelines and informed consents are obtained from the volunteers. At the time of experiment, the volunteer sits on an angle controllable seat, facing the antenna and the adjustable antenna holder. The antenna is connected with the microwave transceiver and data processing computer. A 15 *mm* foam spacer is used in order to fix the distance of the antenna from the volunteer’s head. The backrest of the chair provides support to the volunteer’s back and holds the head in a reasonably fixed position. A light reference head band is utilized to follow-up the investigation plane. As each volunteer has different head appearances, the shape and size of the head is also measured by assessing the reference head band plane. The total power transmitted by the prototype system is 0 dBm. The data from the head of the volunteer are acquired in 1–2 minutes from various angles around the head. The data is then stored and analysed by using the proposed algorithm based on the model of effective head permittivity. The results of the healthy volunteers are presented in Fig. 7.

## Additional Information

**How to cite this article**: Mobashsher, A. T. *et al*. Portable Wideband Microwave Imaging System for Intracranial Hemorrhage Detection Using Improved Back-projection Algorithm with Model of Effective Head Permittivity. *Sci. Rep.* **6**, 20459; doi: 10.1038/srep20459 (2016).

## Supplementary Material

Supplementary Information

## Figures and Tables

**Table 1:** Quantitative metrics indicating the reconstructed image quality.

Effective Permittivity	Simulation model (Fig. 2(g–l))			Large, deep (Fig. 4(a–f))			Small, mid (Fig. 4(g–l))		
	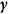	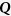	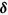 (*mm*)	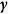	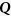	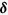 (*mm*)	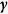	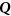	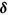 (*mm*)
with ε_avg_ = 30	0.37	0.26	103.53	0.75	1.35	19.81	0.99	3.98	9.7
with ε_avg_ = 35	0.24	0.11	104.37	0.91	3.94	15.7	1.03	5.11	6.02
with ε_avg_ = 40	0.28	0.34	109.08	1.07	6.5	4.3	1.02	5.8	3.64
with ε_avg_ = 45	0.71	0.97	103.96	1.12	6.62	5.15	1.04	6.11	**1.11**
with ε_avg_ = 50	0.87	2.63	99.11	0.99	5.6	11.42	1.05	5.92	3.64
Proposed model (SNR = 30 dB)	**1.39**	**5.83**	**0.71**	**1.17**	**9.9**	**3.54**	**1.25**	**11.4**	2.06
SNR = 20 dB	1.34	5.74	1.58	1.2	9.8	3.9	1.16	11.4	2.7
SNR = 10 dB	1.26	5.34	0.71	1.1	7.1	5.1	1.08	11	4.6
SNR = 5 dB	0.99	4.71	118	0.86	1.8	53.2	0.77	3.8	59
