# Supplementary material for: Portable Wideband Microwave Imaging System for Intracranial Hemorrhage Detection Using Improved Back-projection Algorithm with Model of Effective Head Permittivity
Source: Sci Rep. 2016 Feb 4;6:20459. doi: 10.1038/srep20459 (PMC4740863; doi:10.1038/srep20459)
Supplement: Supplementary Information [file srep20459-s1.pdf]

Scientific Reports

Supplementary Information

Portable Wideband Microwave Imaging System for Intracranial Hemorrhage Detection Using Improved Back-projection Algorithm with Model of Effective Head Permittivity

Ahmed Toaha Mobashsher[1,]*, A. Mahmoud[2], and A.M. Abbosh[1]

[1]School of ITEE, The University of Queensland, St Lucia, 4072, Brisbane, Australia

[2]School of Medicine, Griffith University, Gold Coast, 4215, Australia

*Email: a.mobashsher@uq.edu.au

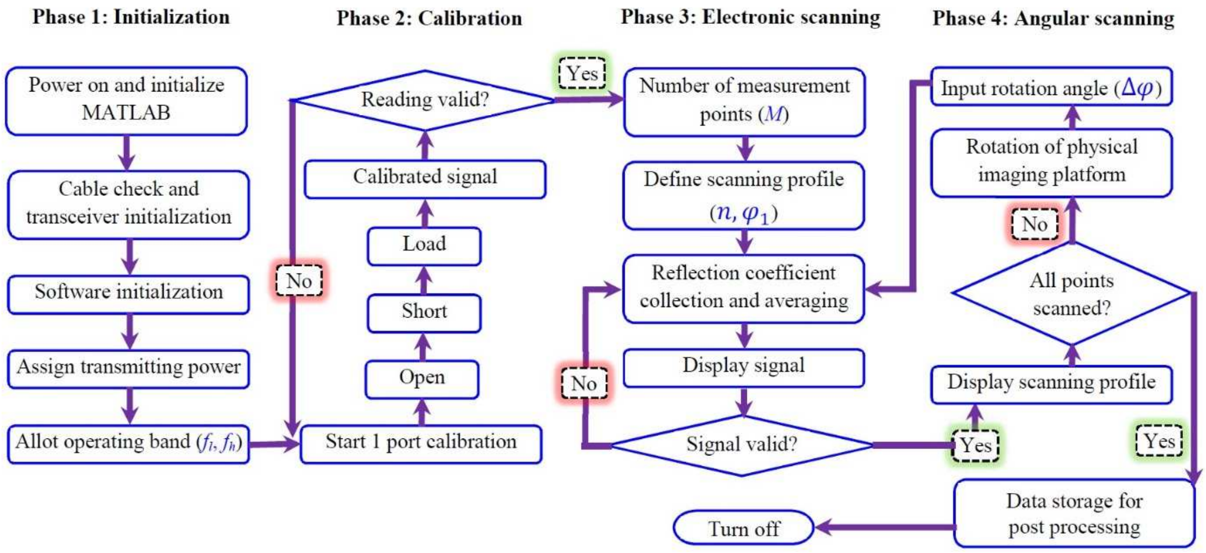

Supplementary Figure S1. The step-by-step flow diagram of the data collection and storage process using a customized controlling software system which creates interfaces between the utilized devices and computer. The data collection process is done in four main stages: 1) initialization, 2) calibration, 3) frequency scanning and 4) angular scanning. As the MATLAB environment is initialized in the computer, the data acquisition devices and controlling software are started along with a check of the connections. Then, a transmitting power of 0 dBm and lowest (f_l = 0.75 GHz) and highest (f_h = 2.55 GHz) frequencies of the operating band of the system are defined. Afterwards, one port calibration process is commenced where the microwave transceiver, Agilent N7081A is calibrated with the help of standard open, short and matching load technique. Then, the number of measurement points, M and equidistant-equiangular scanning profile are defined in the software interface. The reflection coefficient is collected from where the backscattered signal can be extracted. The data are checked for any abnormality, like rapid impulsive change caused from interferences. After this, the scanning profile is checked and in case there are more positions to scan, the imaging platform is rotated by $\Delta\varphi$ angle. The angular input is provided in the controlling software and a combination of frequency and angular scanning is performed until readings from N scanning positions are gathered.

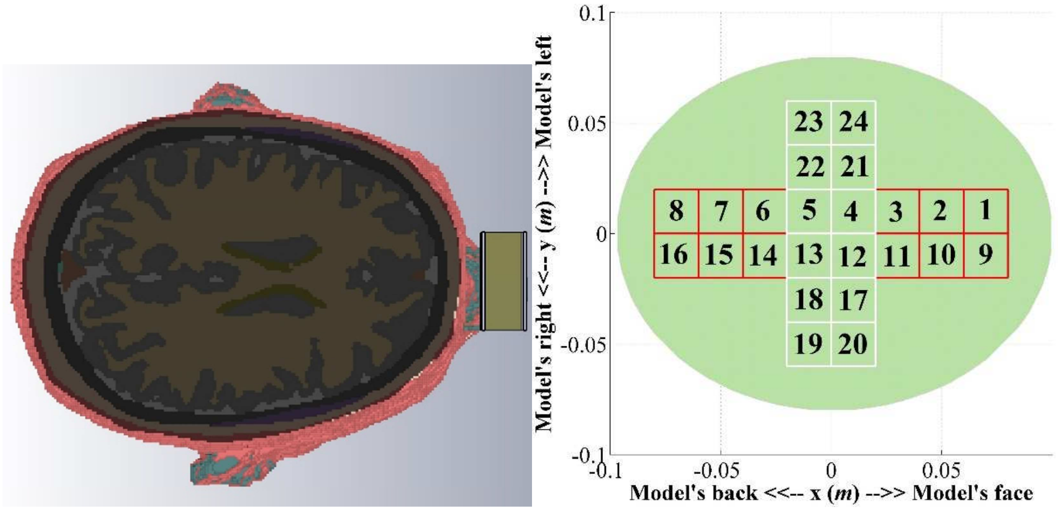

(a) (b)

Supplementary Figure S2. In order to map the accuracy and test the sensitivity of the proposed ICH detection system and the proposed reconstruction algorithm, the cross section of the realistic human head model is divided into 24 individual sectors along the x and y-axes. (a) Illustration of the cross section of a healthy human head model. (b) The sectorial view of the head model indicating positions of different ICH target positions. These target positions are then accordingly defined with ICH properties and simulated in realistic environment. The proposed algorithm is utilized to reconstruct the images. The results are discussed in the manuscript and later in this supplementary section.

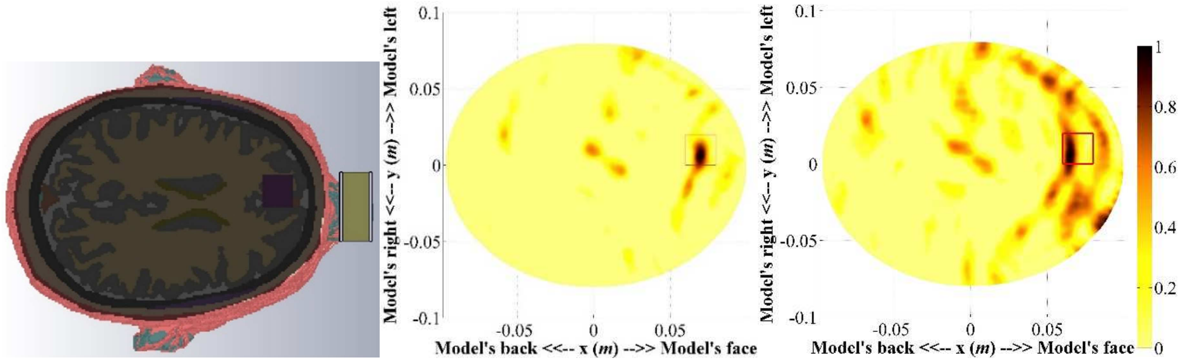

(a) (b) (c)

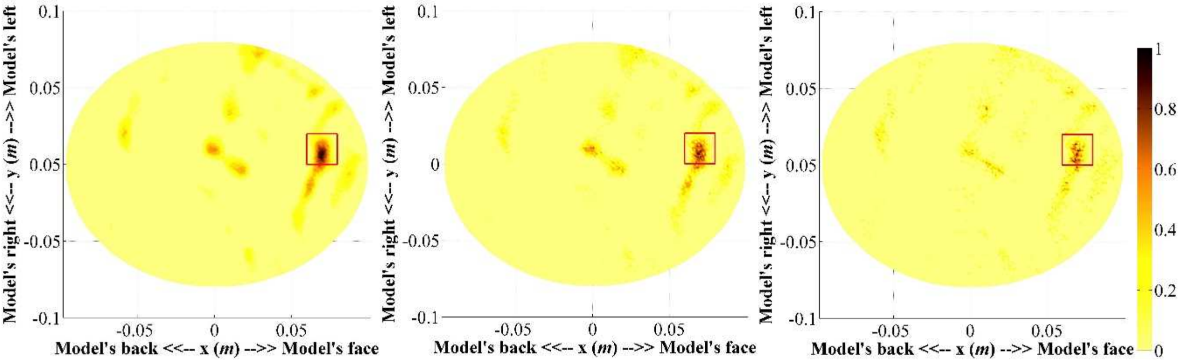

(d) (e) (f)

Supplementary Figure S3. (a) The representative scenario of realistic ICH affected human head at position 1 with the antenna placed in front of the head model. (b) The reconstructed image (SNR = 30 dB) of the head cross section using the proposed algorithm relying on the model of effective head permittivity. (c) Resulted image of the head cross section at the same situation and using the same dataset, but using the existing algorithm based on

constant effective head permittivity of ε_{eff} = 45. The reconstructed image of head cross section utilizing the improved back-projection algorithm at (d) SNR = 20 dB, (e) SNR = 10 dB and (f) SNR = 5 dB.

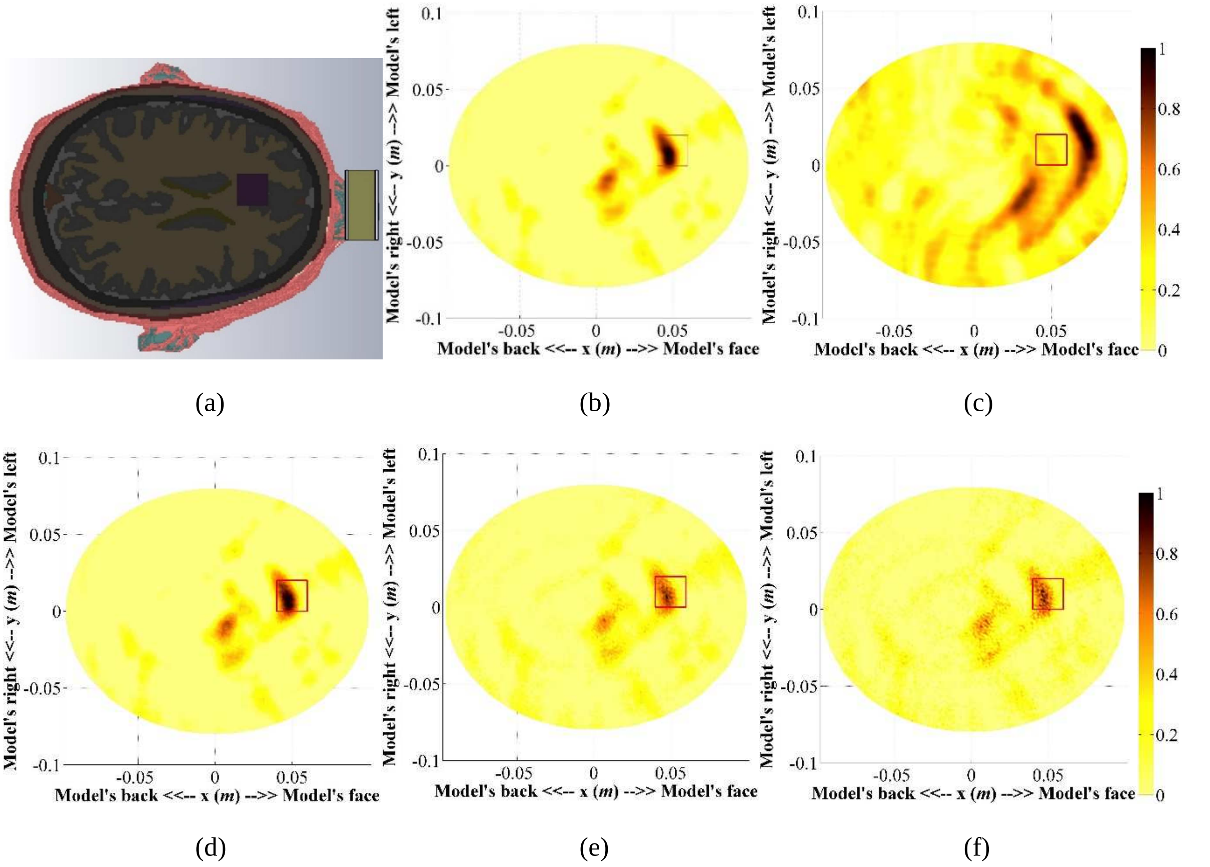

Supplementary Figure S4. (a) The representative scenario of realistic ICH affected human head at position 2 with the antenna placed in front of the head model. (b) The reconstructed image (SNR = 30 dB) of the head cross section using the proposed algorithm relying on the model of effective head permittivity. (c) Resulted image of the head cross section at the same situation and using the same dataset, but using the existing algorithm based on constant effective head permittivity of ε_{eff} = 45. The reconstructed image of head cross section utilizing the improved back-projection algorithm at (d) SNR = 20 dB, (e) SNR = 10 dB and (f) SNR = 5 dB.

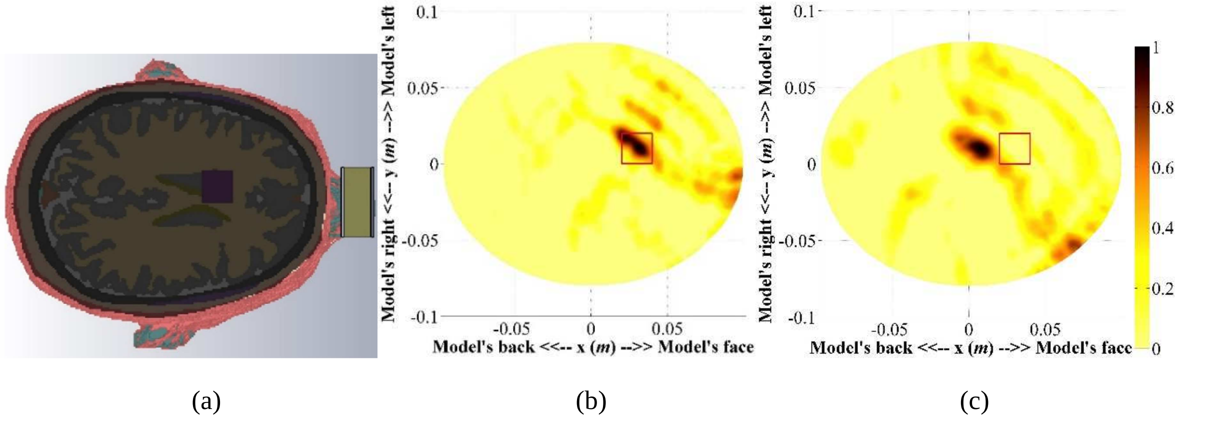

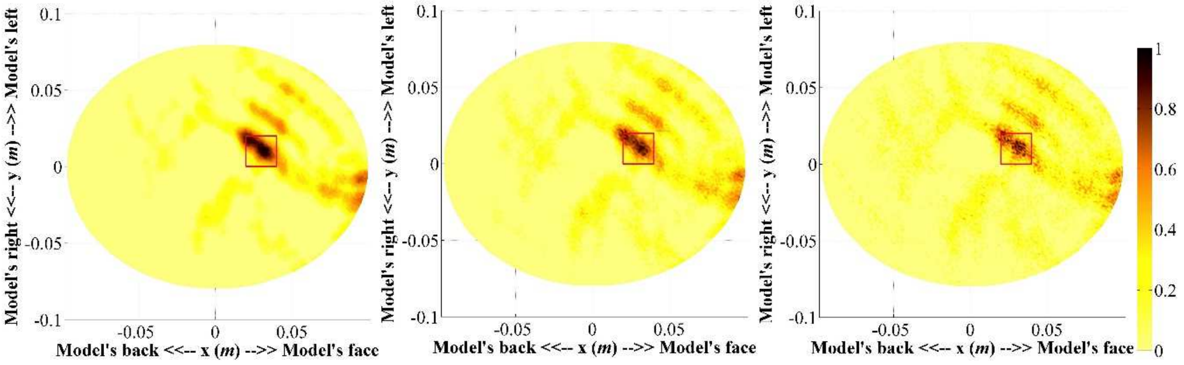

(d) (e) (f)

Supplementary Figure S5. (a) The representative scenario of realistic ICH affected human head at position 3 with the antenna placed in front of the head model. (b) The reconstructed image (SNR = 30 dB) of the head cross section using the proposed algorithm relying on the model of effective head permittivity. (c) Resulted image of the head cross section at the same situation and using the same dataset, but using the existing algorithm based on constant effective head permittivity of ε_{eff} = 45. The reconstructed image of head cross section utilizing the improved back-projection algorithm at (d) SNR = 20 dB, (e) SNR = 10 dB and (f) SNR = 5 dB.

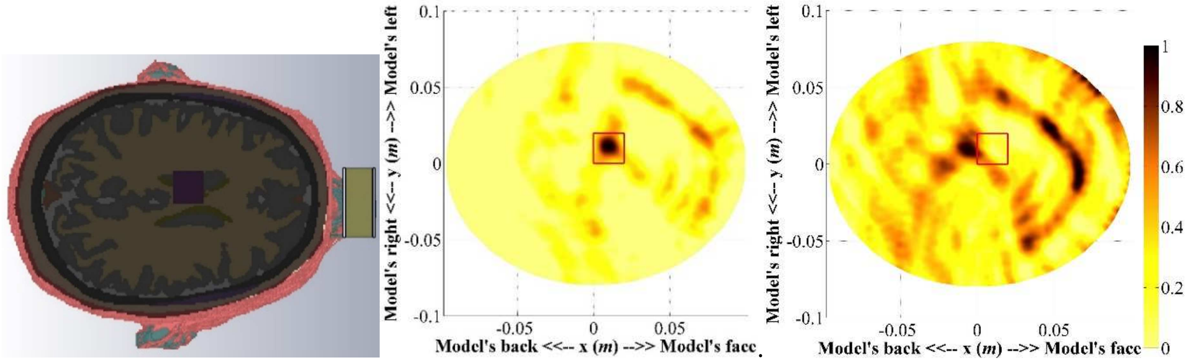

(a) (b) (c)

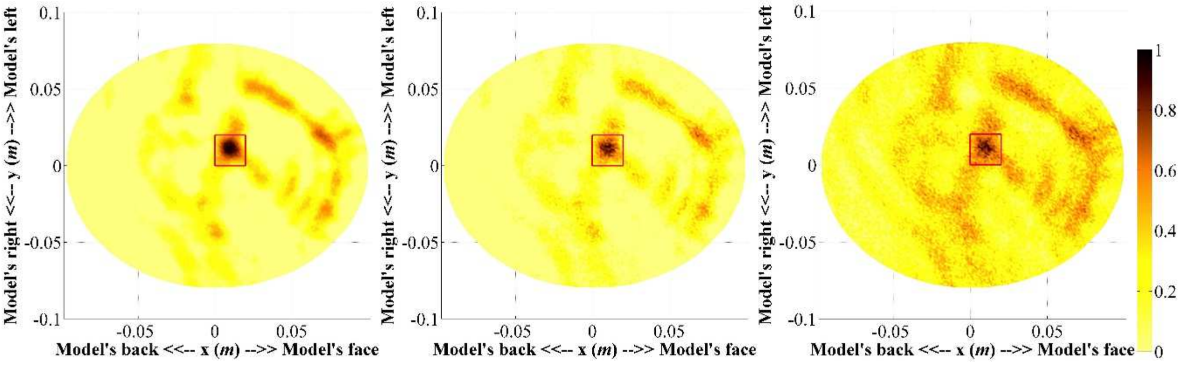

(d) (e) (f)

Supplementary Figure S6. (a) The representative scenario of realistic ICH affected human head at position 4 with the antenna placed in front of the head model. (b) The reconstructed image (SNR = 30 dB) of the head cross section using the proposed algorithm relying on the model of effective head permittivity. (c) Resulted image of the head cross section at the same situation and using the same dataset, but using the existing algorithm based on constant effective head permittivity of ε_{eff} = 45. The reconstructed image of head cross section utilizing the improved back-projection algorithm at (d) SNR = 20 dB, (e) SNR = 10 dB and (f) SNR = 5 dB.

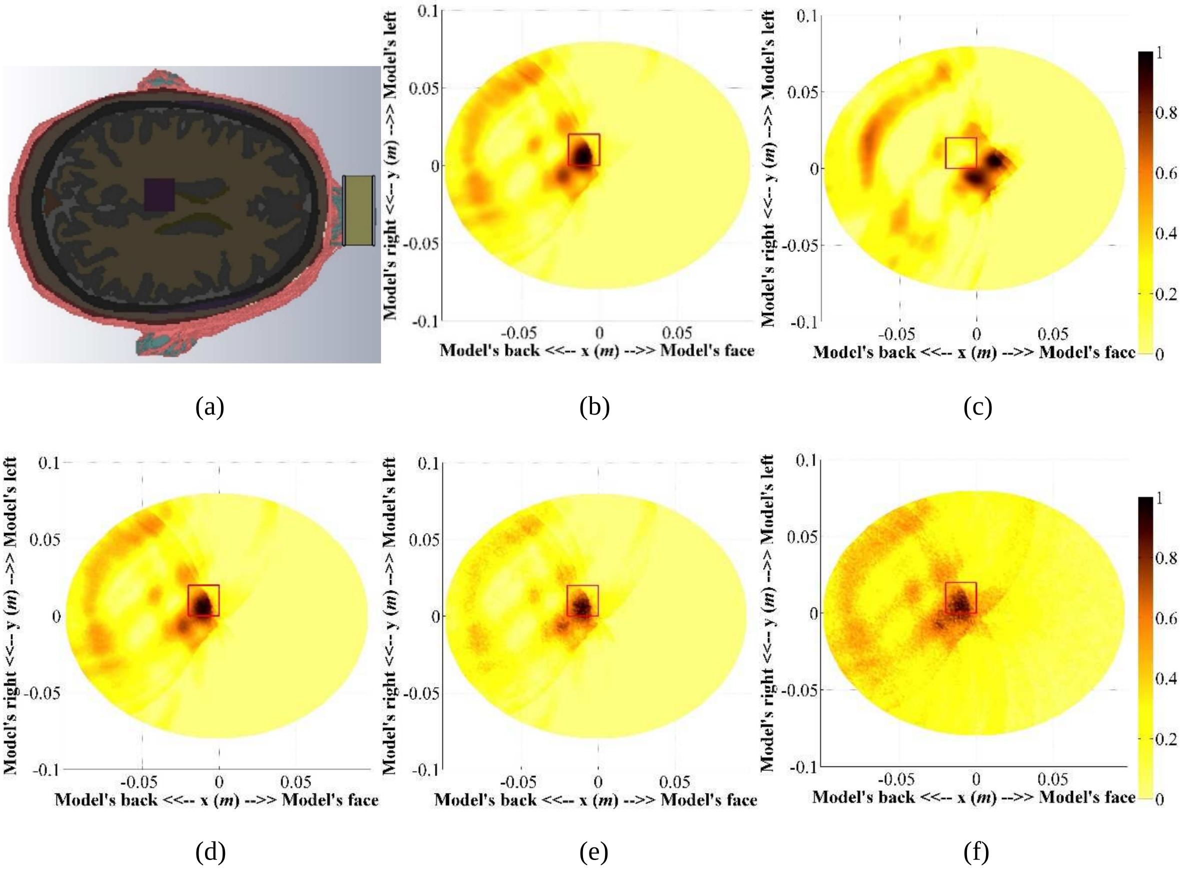

Supplementary Figure S7. (a) The representative scenario of realistic ICH affected human head at position 5 with the antenna placed in front of the head model. (b) The reconstructed image (SNR = 30 dB) of the head cross section using the proposed algorithm relying on the model of effective head permittivity. (c) Resulted image of the head cross section at the same situation and using the same dataset, but using the existing algorithm based on constant effective head permittivity of ε_{eff} = 45. The reconstructed image of head cross section utilizing the improved back-projection algorithm at (d) SNR = 20 dB, (e) SNR = 10 dB and (f) SNR = 5 dB.

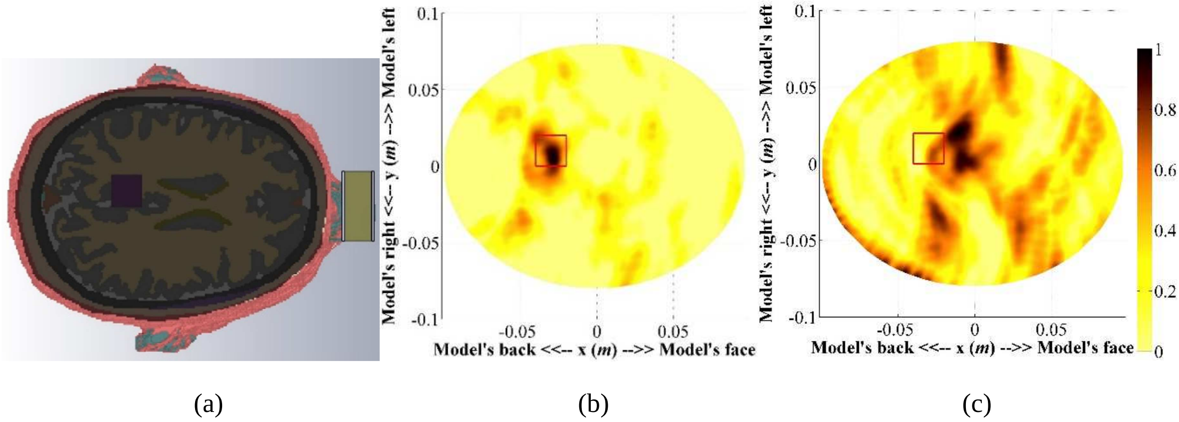

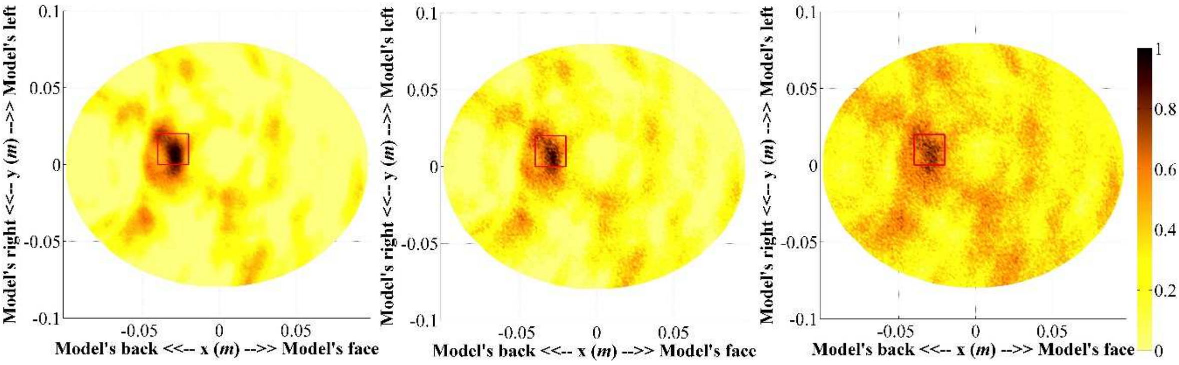

(d) (e) (f)

Supplementary Figure S8. (a) The representative scenario of realistic ICH affected human head at position 6 with the antenna placed in front of the head model. (b) The reconstructed image (SNR = 30 dB) of the head cross section using the proposed algorithm relying on the model of effective head permittivity. (c) Resulted image of the head cross section at the same situation and using the same dataset, but using the existing algorithm based on constant effective head permittivity of ε_{eff} = 45. The reconstructed image of head cross section utilizing the improved back-projection algorithm at (d) SNR = 20 dB, (e) SNR = 10 dB and (f) SNR = 5 dB.

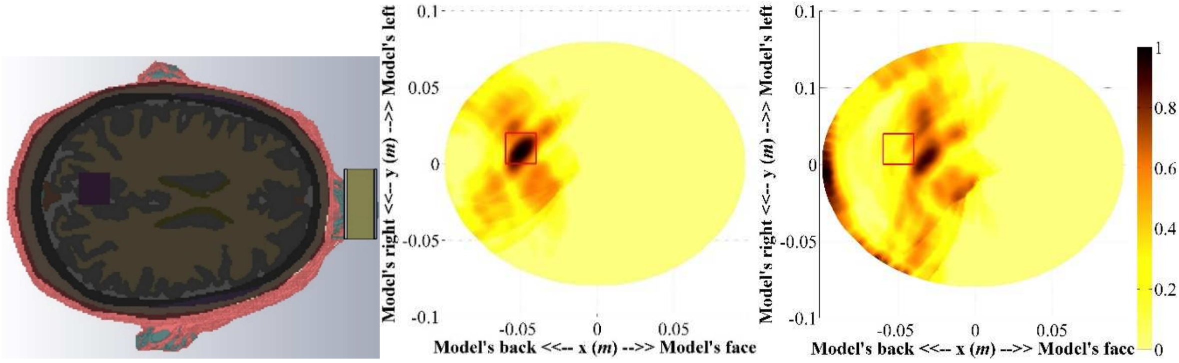

(a) (b) (c)

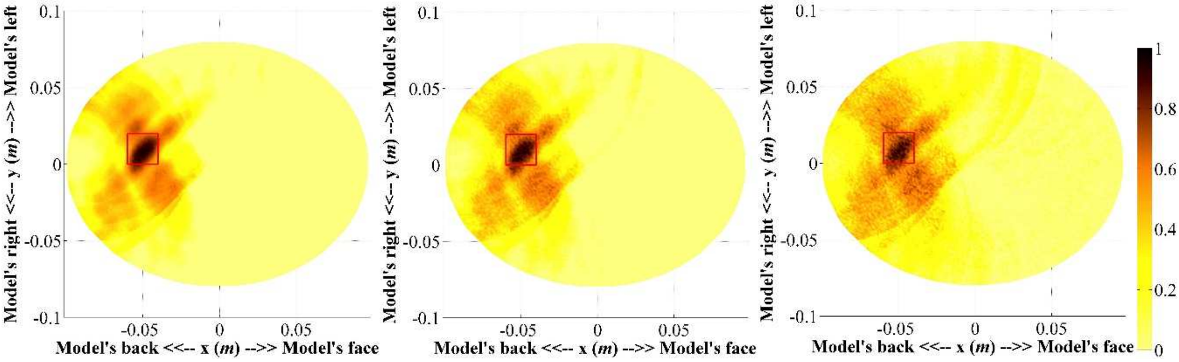

(d) (e) (f)

Supplementary Figure S9. (a) The representative scenario of realistic ICH affected human head at position 7 with the antenna placed in front of the head model. (b) The reconstructed image (SNR = 30 dB) of the head cross section using the proposed algorithm relying on the model of effective head permittivity. (c) Resulted image of the head cross section at the same situation and using the same dataset, but using the existing algorithm based on constant effective head permittivity of ε_{eff} = 45. The reconstructed image of head cross section utilizing the improved back-projection algorithm at (d) SNR = 20 dB, (e) SNR = 10 dB and (f) SNR = 5 dB.

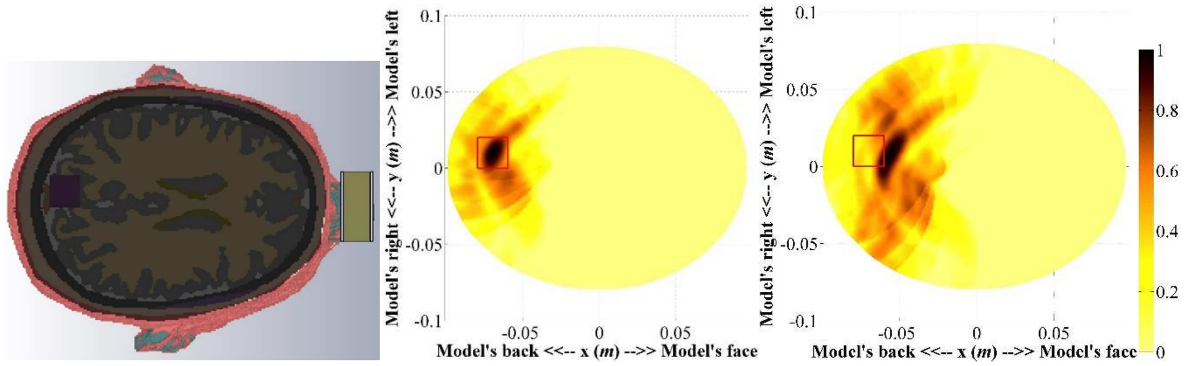

(a) (b) (c)

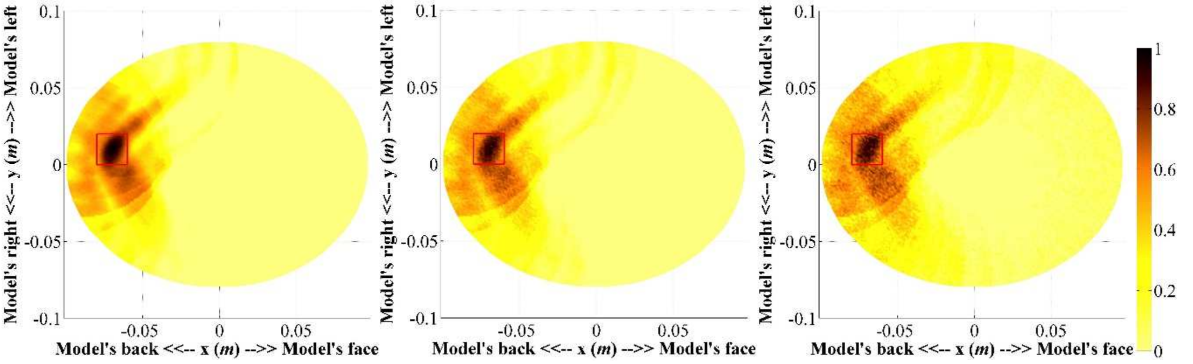

(d) (e) (f)

Supplementary Figure S10. (a) The representative scenario of realistic ICH affected human head at position 8 with the antenna placed in front of the head model. (b) The reconstructed image (SNR = 30 dB) of the head cross section using the proposed algorithm relying on the model of effective head permittivity. (c) Resulted image of the head cross section at the same situation and using the same dataset, but using the existing algorithm based on constant effective head permittivity of ε_{eff} = 45. The reconstructed image of head cross section utilizing the improved back-projection algorithm at (d) SNR = 20 dB, (e) SNR = 10 dB and (f) SNR = 5 dB.

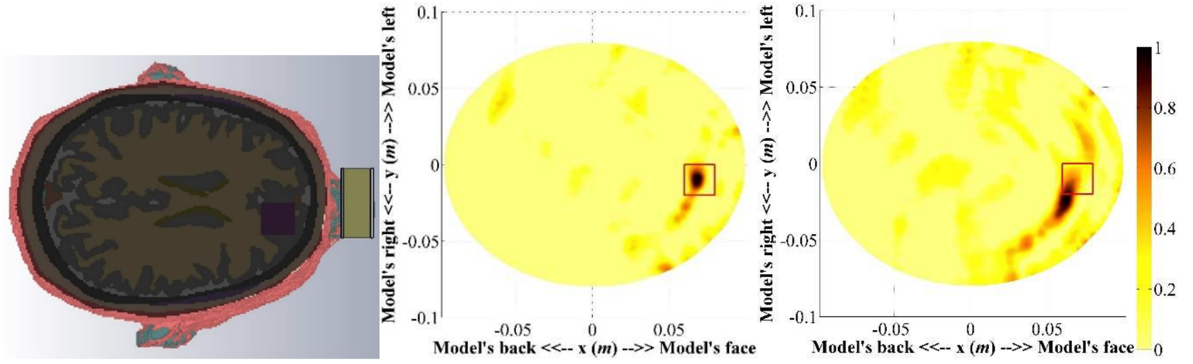

(a) (b) (c)

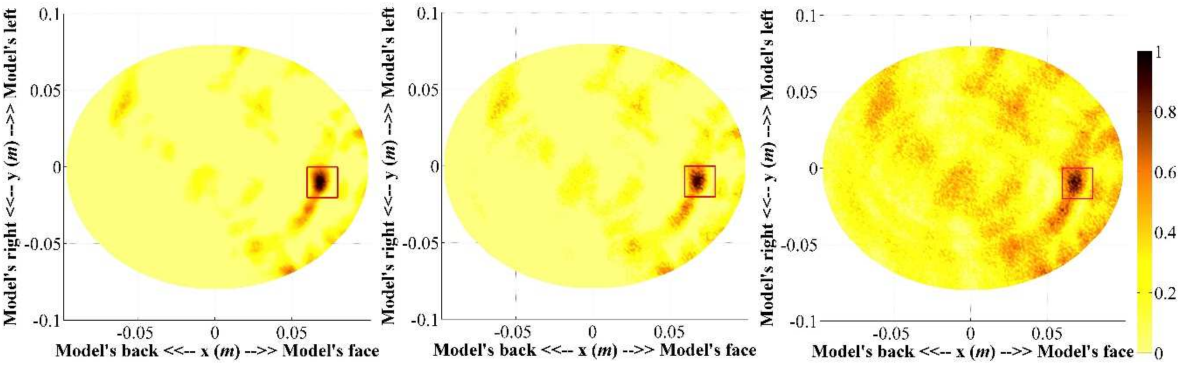

(d) (e) (f)

Supplementary Figure S11. (a) The representative scenario of realistic ICH affected human head at position 9 with the antenna placed in front of the head model. (b) The reconstructed image (SNR = 30 dB) of the head cross section using the proposed algorithm relying on the model of effective head permittivity. (c) Resulted image of the head cross section at the same situation and using the same dataset, but using the existing algorithm based on constant effective head permittivity of ε_{eff} = 45. The reconstructed image of head cross section utilizing the improved back-projection algorithm at (d) SNR = 20 dB, (e) SNR = 10 dB and (f) SNR = 5 dB.

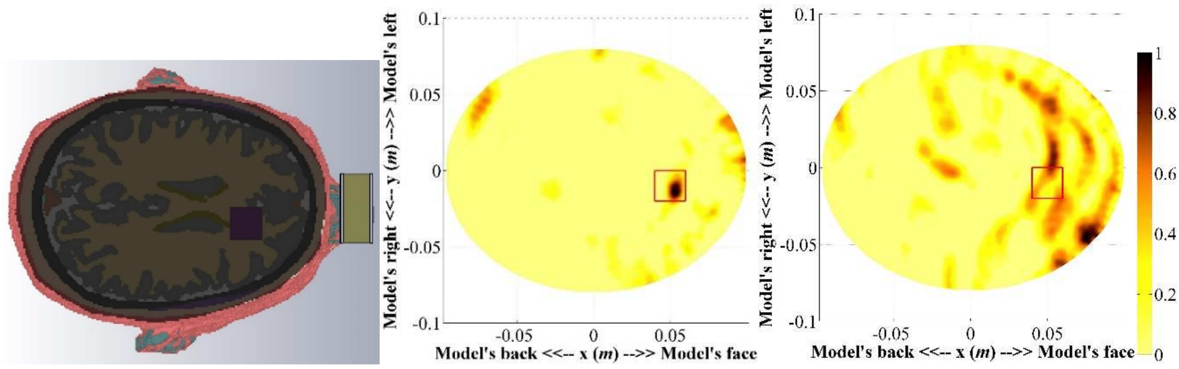

(a) (b) (c)

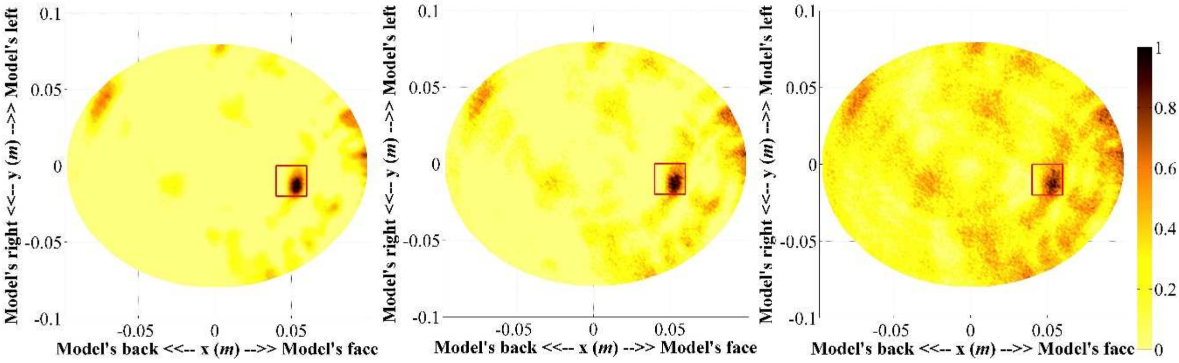

(d) (e) (f)

Supplementary Figure S12. (a) The representative scenario of realistic ICH affected human head at position 10 with the antenna placed in front of the head model. (b) The reconstructed image (SNR = 30 dB) of the head cross section using the proposed algorithm relying on the model of effective head permittivity. (c) Resulted image of the head cross section at the same situation and using the same dataset, but using the existing algorithm based on constant effective head permittivity of ε_{eff} = 45. The reconstructed image of head cross section utilizing the improved back-projection algorithm at (d) SNR = 20 dB, (e) SNR = 10 dB and (f) SNR = 5 dB.

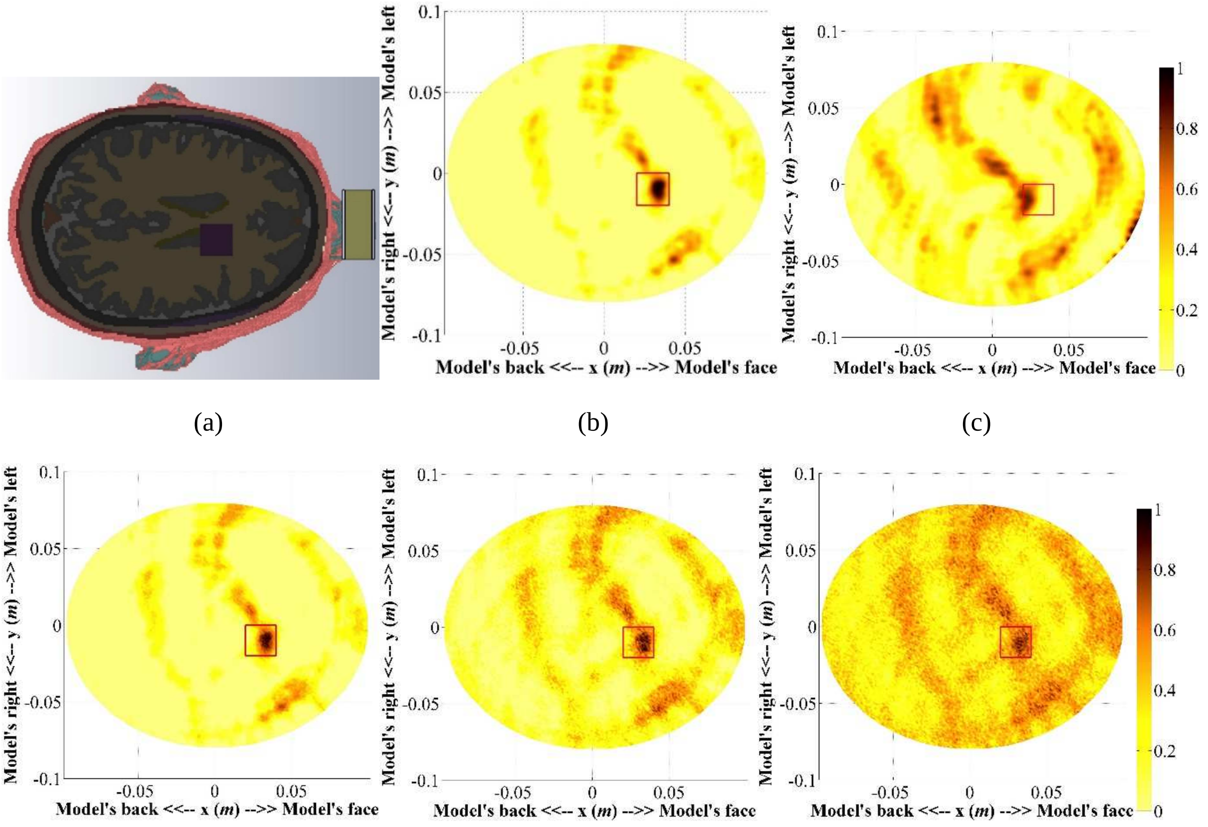

(a) (b) (c)

(d) (e) (f)

Supplementary Figure S13. (a) The representative scenario of realistic ICH affected human head at position 11 with the antenna placed in front of the head model. (b) The reconstructed image (SNR = 30 dB) of the head cross section using the proposed algorithm relying on the model of effective head permittivity. (c) Resulted image of the head cross section at the same situation and using the same dataset, but using the existing algorithm based on constant effective head permittivity of ε_{eff} = 45. The reconstructed image of head cross section utilizing the improved back-projection algorithm at (d) SNR = 20 dB, (e) SNR = 10 dB and (f) SNR = 5 dB.

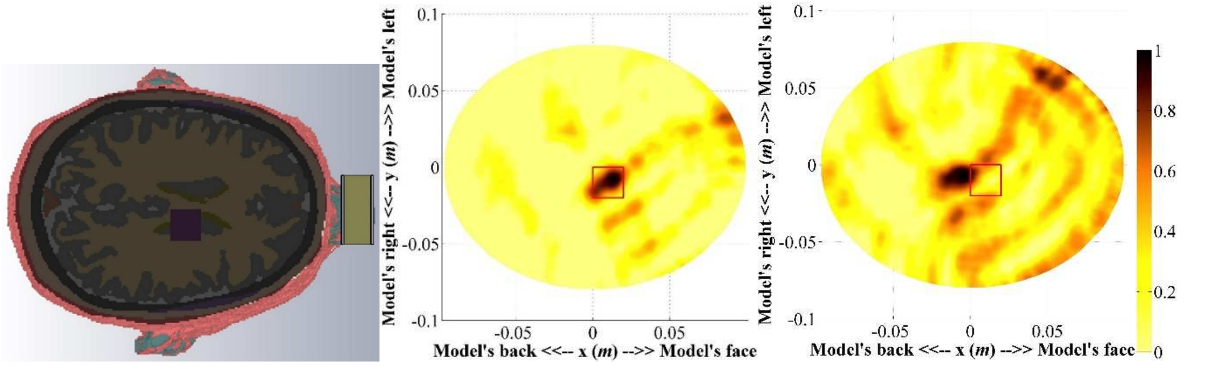

(a) (b) (c)

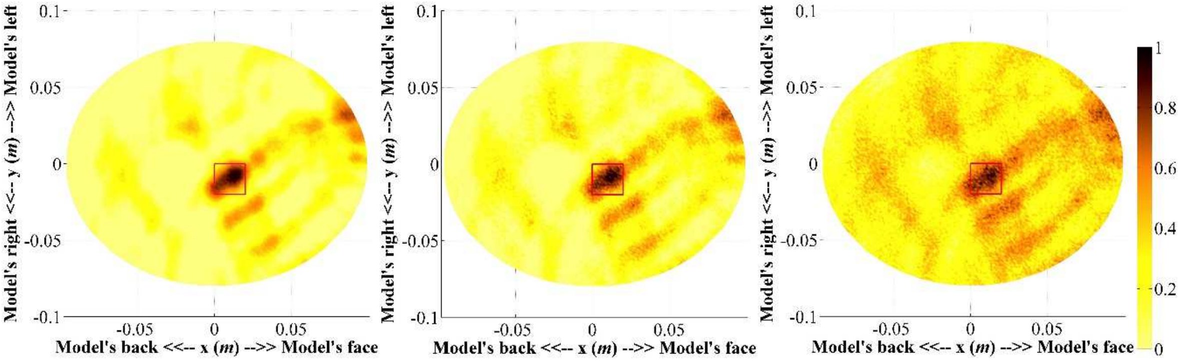

(d) (e) (f)

Supplementary Figure S14. (a) The representative scenario of realistic ICH affected human head at position 12 with the antenna placed in front of the head model. (b) The reconstructed image (SNR = 30 dB) of the head cross section using the proposed algorithm relying on the model of effective head permittivity. (c) Resulted image of the head cross section at the same situation and using the same dataset, but using the existing algorithm based on constant effective head permittivity of ε_{eff} = 45. The reconstructed image of head cross section utilizing the improved back-projection algorithm at (d) SNR = 20 dB, (e) SNR = 10 dB and (f) SNR = 5 dB.

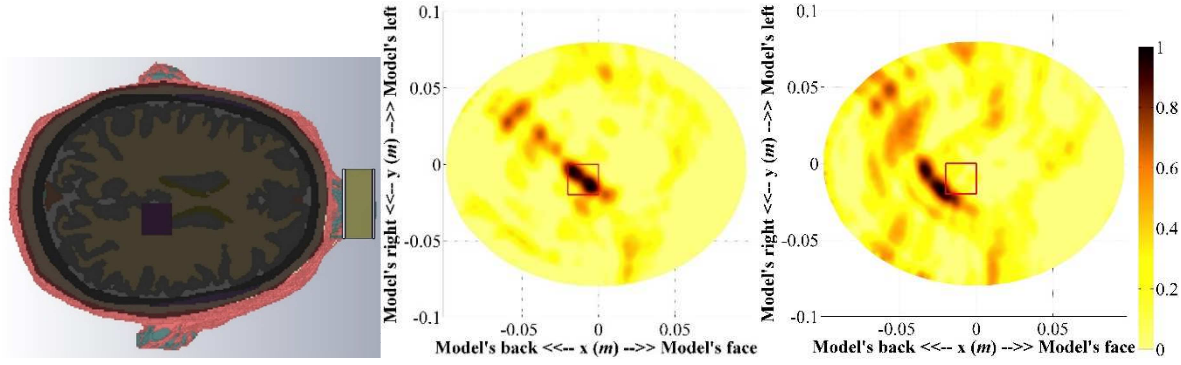

(a) (b) (c)

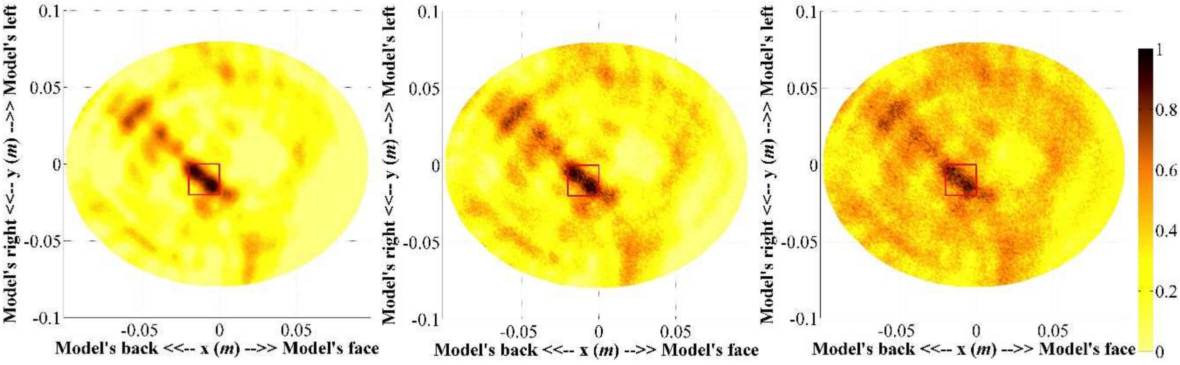

(d) (e) (f)

Supplementary Figure S15. (a) The representative scenario of realistic ICH affected human head at position 13 with the antenna placed in front of the head model. (b) The reconstructed image (SNR = 30 dB) of the head cross section using the proposed algorithm relying on the model of effective head permittivity. (c) Resulted image of the head cross section at the same situation and using the same dataset, but using the existing algorithm based on constant effective head permittivity of ε_{eff} = 45. The reconstructed image of head cross section utilizing the improved back-projection algorithm at (d) SNR = 20 dB, (e) SNR = 10 dB and (f) SNR = 5 dB.

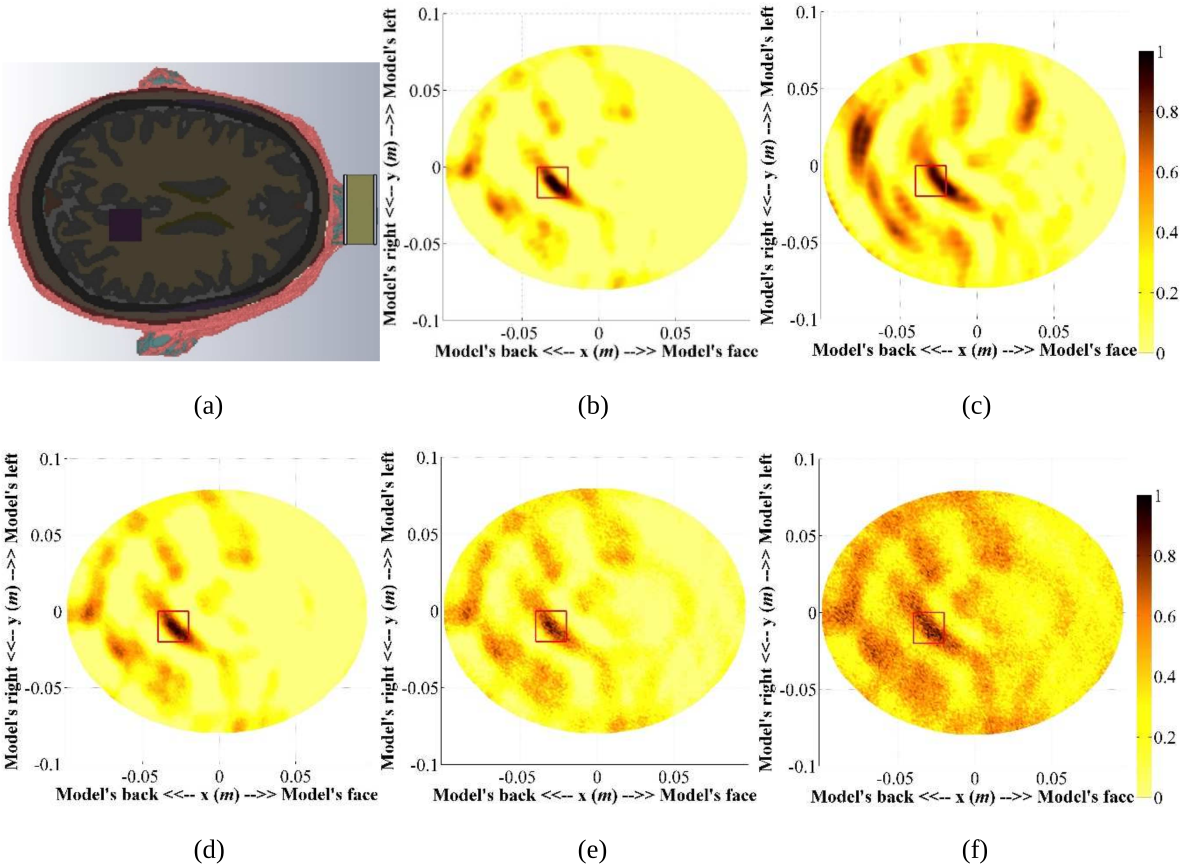

Supplementary Figure S16. (a) The representative scenario of realistic ICH affected human head at position 14 with the antenna placed in front of the head model. (b) The reconstructed image (SNR = 30 dB) of the head cross section using the proposed algorithm relying on the model of effective head permittivity. (c) Resulted image of the head cross section at the same situation and using the same dataset, but using the existing algorithm based on constant effective head permittivity of ε_{eff} = 45. The reconstructed image of head cross section utilizing the improved back-projection algorithm at (d) SNR = 20 dB, (e) SNR = 10 dB and (f) SNR = 5 dB.

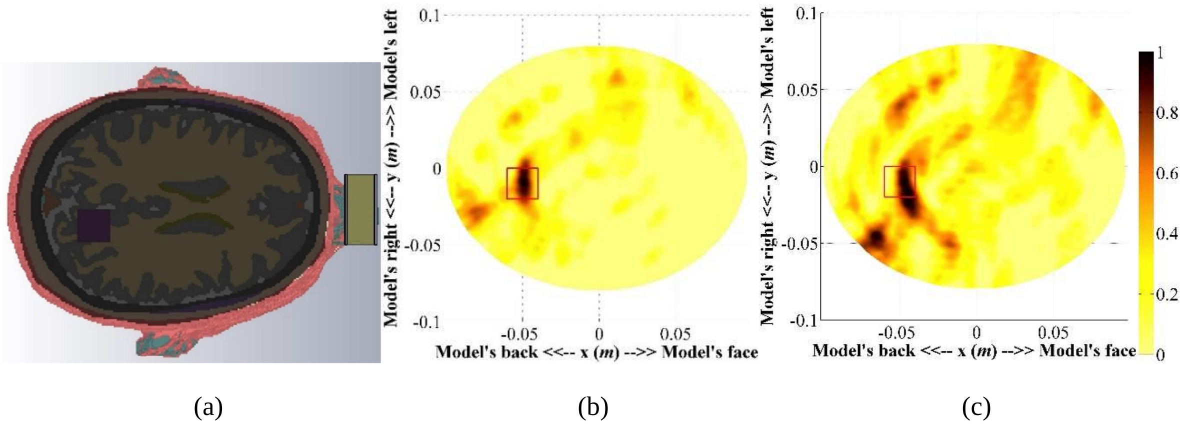

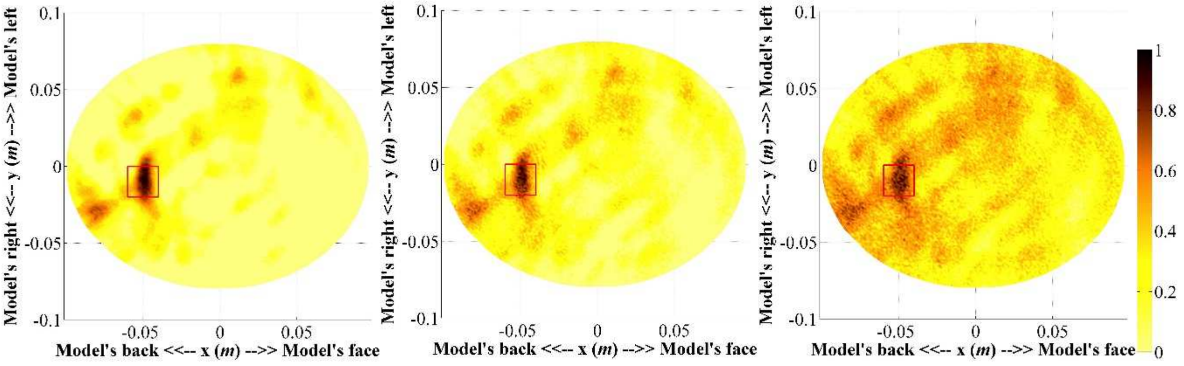

(d) (e) (f)

Supplementary Figure S17. (a) The representative scenario of realistic ICH affected human head at position 15 with the antenna placed in front of the head model. (b) The reconstructed image (SNR = 30 dB) of the head cross section using the proposed algorithm relying on the model of effective head permittivity. (c) Resulted image of the head cross section at the same situation and using the same dataset, but using the existing algorithm based on constant effective head permittivity of ε_{eff} = 45. The reconstructed image of head cross section utilizing the improved back-projection algorithm at (d) SNR = 20 dB, (e) SNR = 10 dB and (f) SNR = 5 dB.

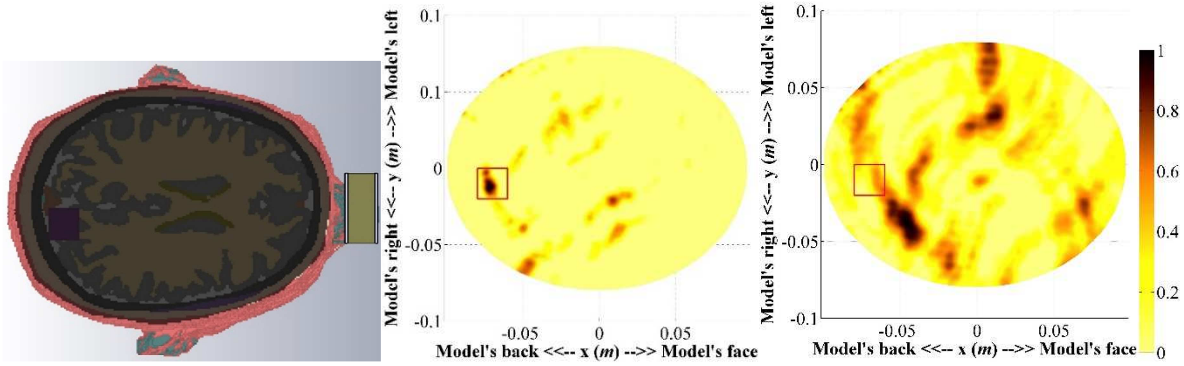

(a) (b) (c)

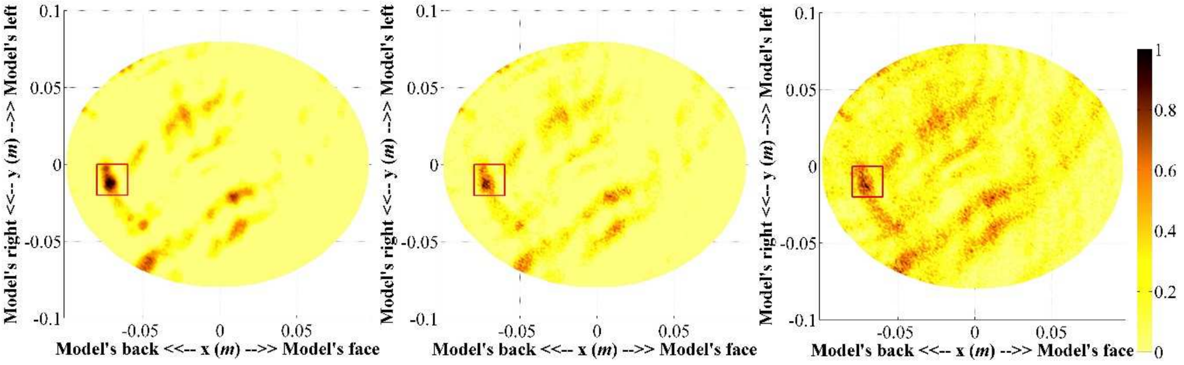

(d) (e) (f)

Supplementary Figure S18. (a) The representative scenario of realistic ICH affected human head at position 16 with the antenna placed in front of the head model. (b) The reconstructed image (SNR = 30 dB) of the head cross section using the proposed algorithm relying on the model of effective head permittivity. (c) Resulted image of the head cross section at the same situation and using the same dataset, but using the existing algorithm based on constant effective head permittivity of ε_{eff} = 45. The reconstructed image of head cross section utilizing the improved back-projection algorithm at (d) SNR = 20 dB, (e) SNR = 10 dB and (f) SNR = 5 dB.

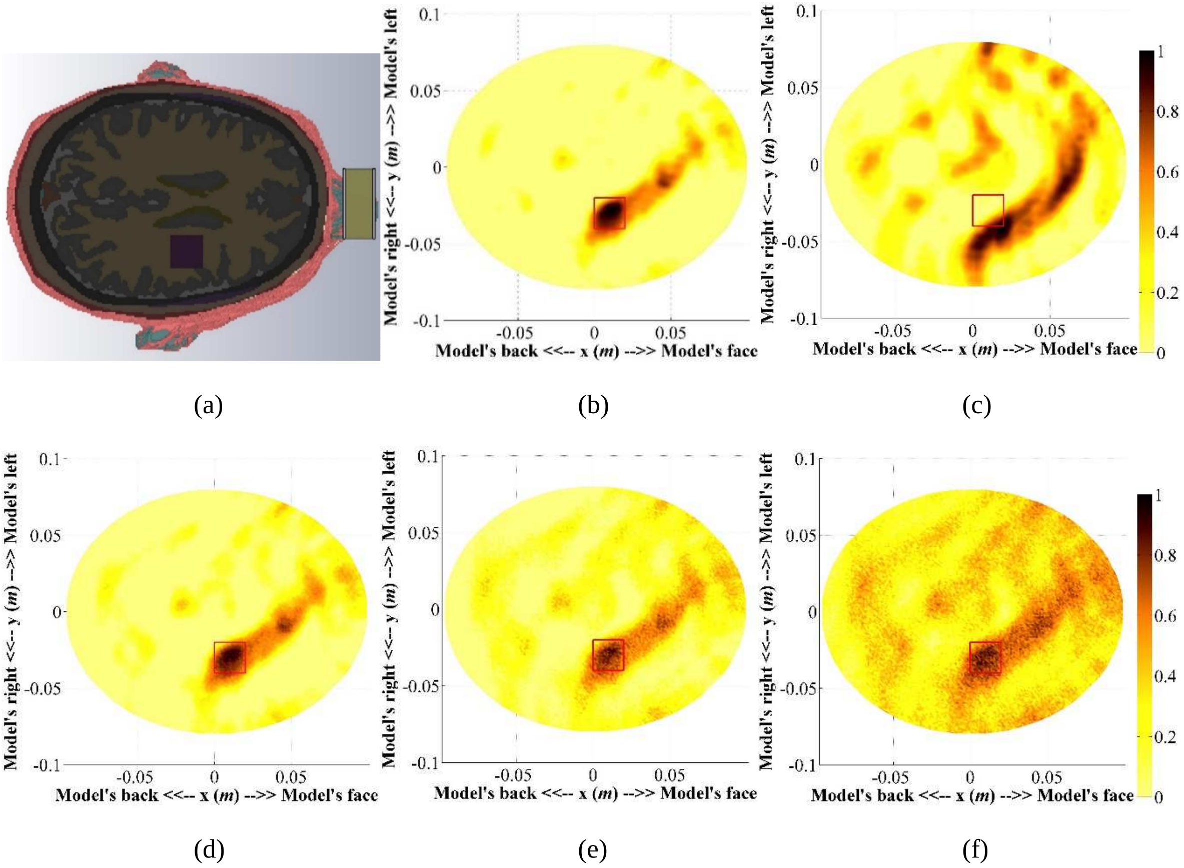

Supplementary Figure S19. (a) The representative scenario of realistic ICH affected human head at position 17 with the antenna placed in front of the head model. (b) The reconstructed image (SNR = 30 dB) of the head cross section using the proposed algorithm relying on the model of effective head permittivity. (c) Resulted image of the head cross section at the same situation and using the same dataset, but using the existing algorithm based on constant effective head permittivity of ε_{eff} = 45. The reconstructed image of head cross section utilizing the improved back-projection algorithm at (d) SNR = 20 dB, (e) SNR = 10 dB and (f) SNR = 5 dB.

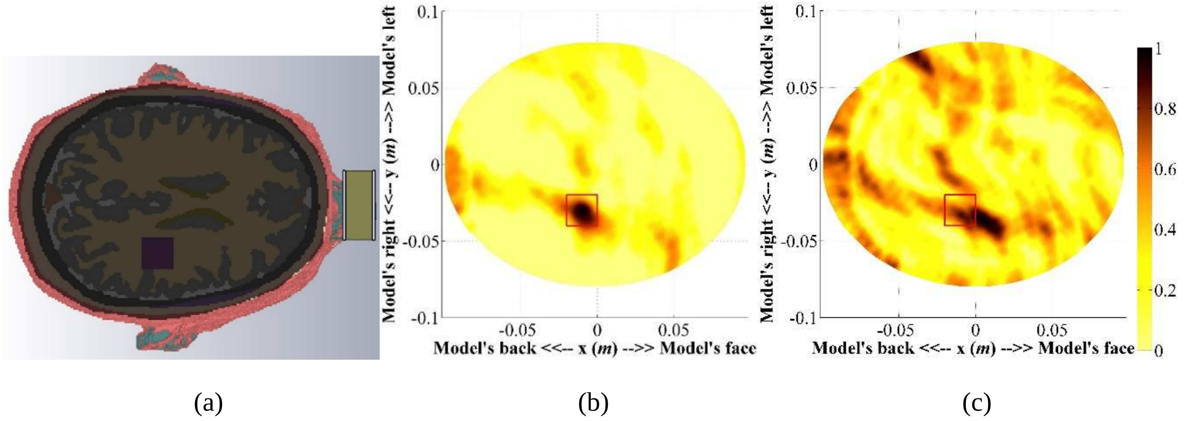

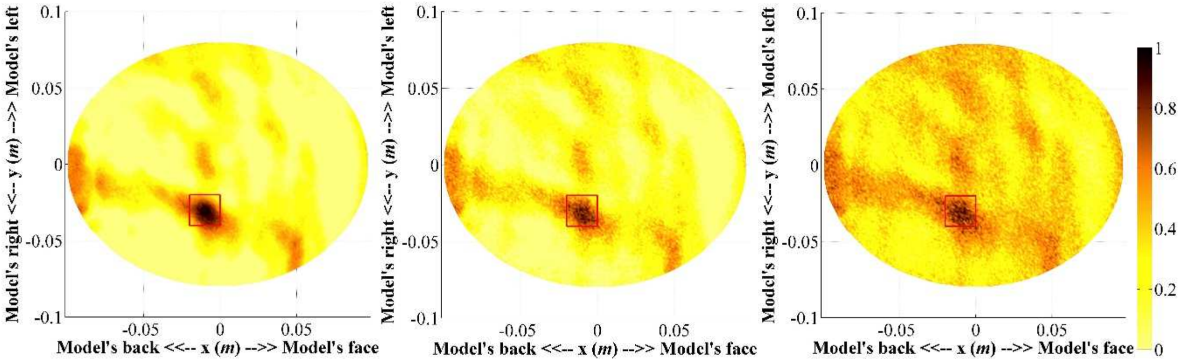

(d) (e) (f)

Supplementary Figure S20. (a) The representative scenario of realistic ICH affected human head at position 18 with the antenna placed in front of the head model. (b) The reconstructed image (SNR = 30 dB) of the head cross section using the proposed algorithm relying on the model of effective head permittivity. (c) Resulted image of the head cross section at the same situation and using the same dataset, but using the existing algorithm based on constant effective head permittivity of ε_{eff} = 45. The reconstructed image of head cross section utilizing the improved back-projection algorithm at (d) SNR = 20 dB, (e) SNR = 10 dB and (f) SNR = 5 dB.

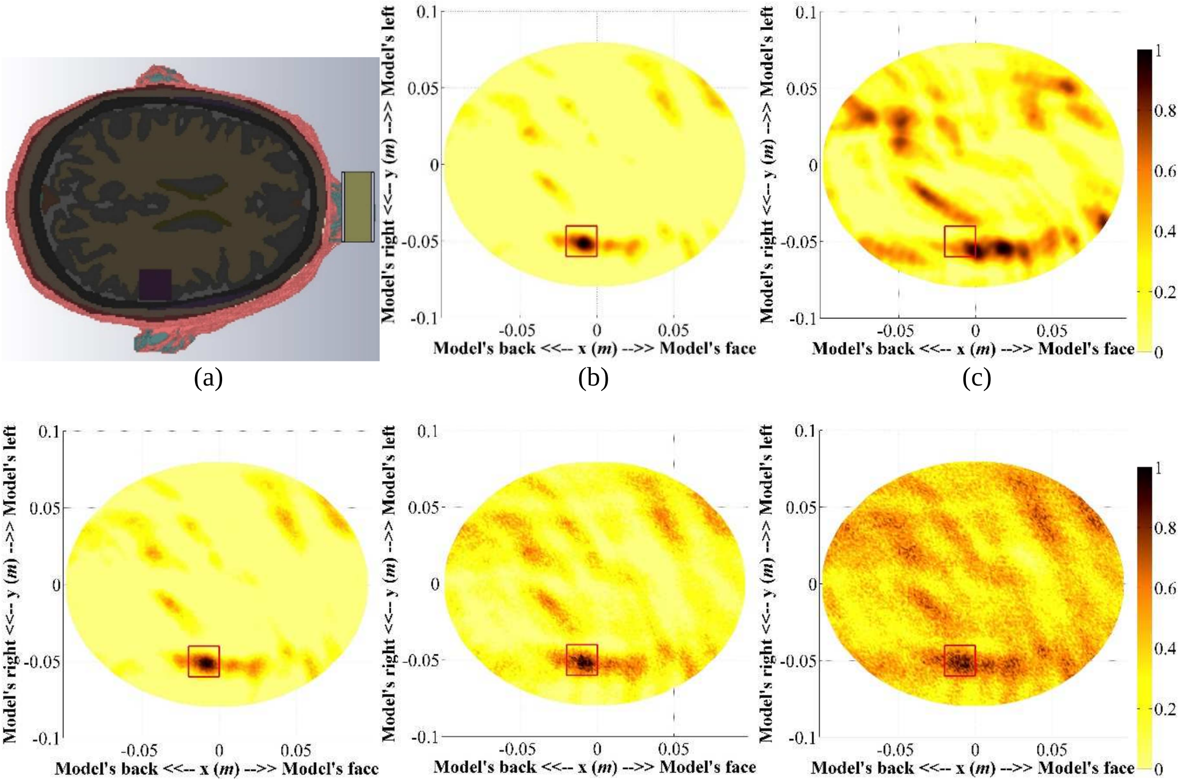

(d) (e) (f)

Supplementary Figure S21. (a) The representative scenario of realistic ICH affected human head at position 19 with the antenna placed in front of the head model. (b) The reconstructed image (SNR = 30 dB) of the head cross section using the proposed algorithm relying on the model of effective head permittivity. (c) Resulted image of the head cross section at the same situation and using the same dataset, but using the existing algorithm based on constant effective head permittivity of ε_{eff} = 45. The reconstructed image of head cross section utilizing the improved back-projection algorithm at (d) SNR = 20 dB, (e) SNR = 10 dB and (f) SNR = 5 dB.

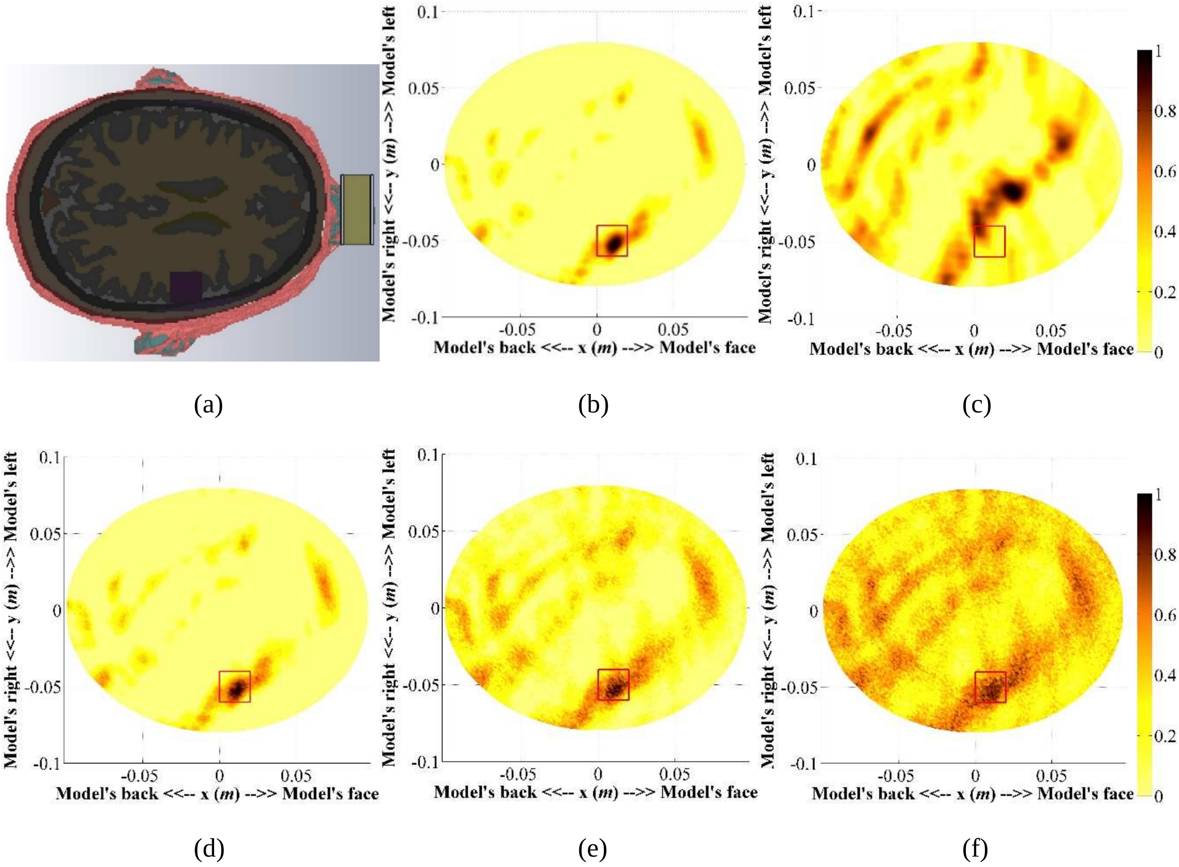

Supplementary Figure S22. (a) The representative scenario of realistic ICH affected human head at position 20 with the antenna placed in front of the head model. (b) The reconstructed image (SNR = 30 dB) of the head cross section using the proposed algorithm relying on the model of effective head permittivity. (c) Resulted image of the head cross section at the same situation and using the same dataset, but using the existing algorithm based on constant effective head permittivity of ε_{eff} = 45. The reconstructed image of head cross section utilizing the improved back-projection algorithm at (d) SNR = 20 dB, (e) SNR = 10 dB and (f) SNR = 5 dB.

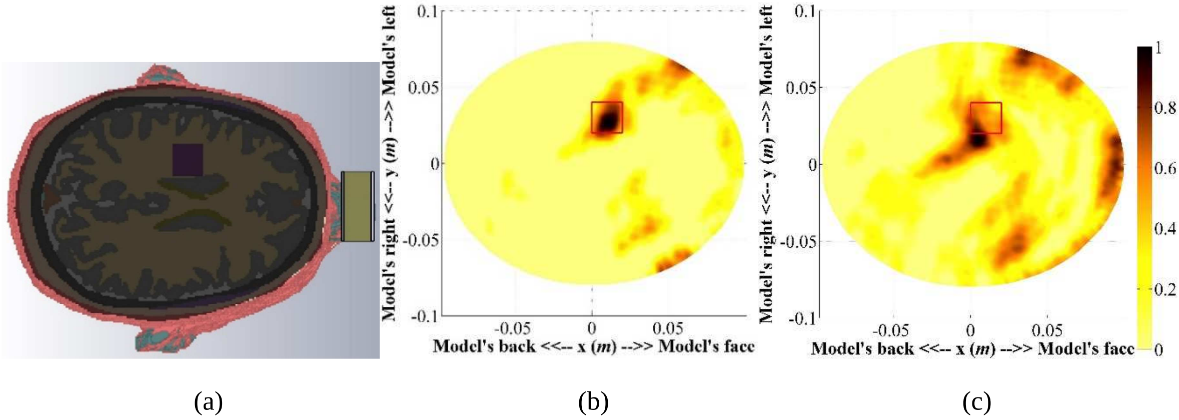

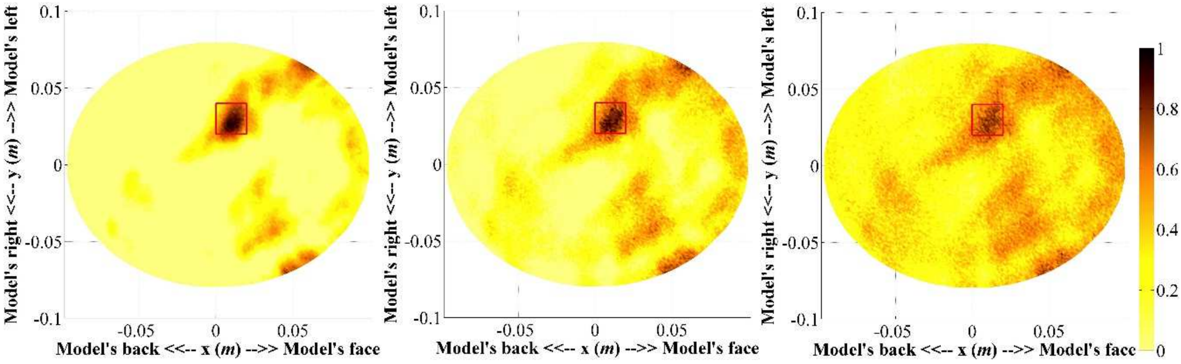

(d) (e) (f)

Supplementary Figure S23. (a) The representative scenario of realistic ICH affected human head at position 21 with the antenna placed in front of the head model. (b) The reconstructed image (SNR = 30 dB) of the head cross section using the proposed algorithm relying on the model of effective head permittivity. (c) Resulted image of the head cross section at the same situation and using the same dataset, but using the existing algorithm based on constant effective head permittivity of ε_{eff} = 45. The reconstructed image of head cross section utilizing the improved back-projection algorithm at (d) SNR = 20 dB, (e) SNR = 10 dB and (f) SNR = 5 dB.

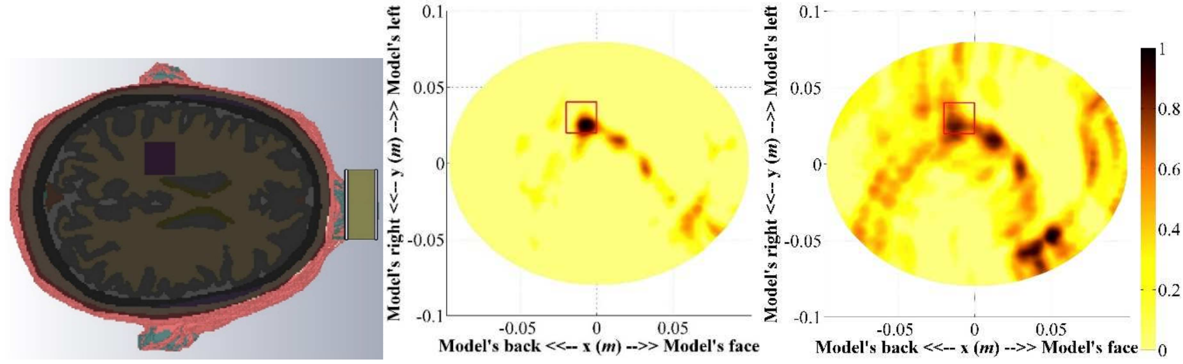

(a) (b) (c)

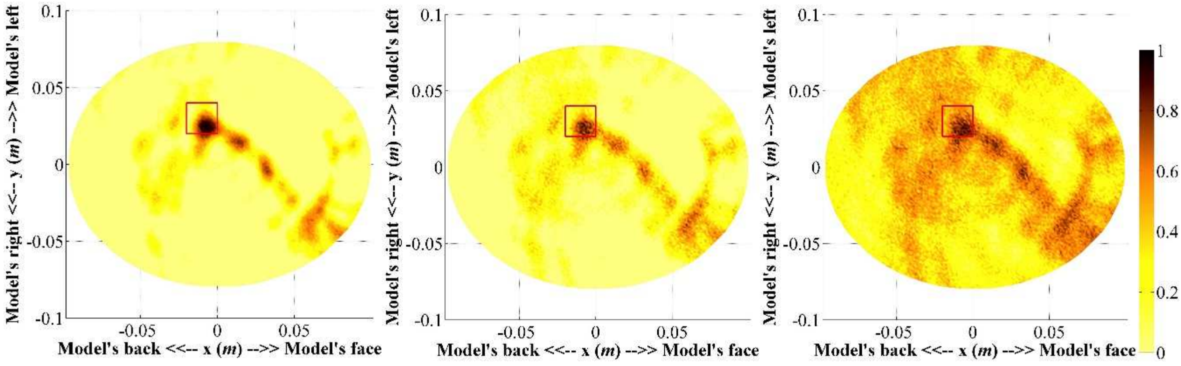

(d) (e) (f)

Supplementary Figure S24. (a) The representative scenario of realistic ICH affected human head at position 22 with the antenna placed in front of the head model. (b) The reconstructed image (SNR = 30 dB) of the head cross section using the proposed algorithm relying on the model of effective head permittivity. (c) Resulted image of the head cross section at the same situation and using the same dataset, but using the existing algorithm based on constant effective head permittivity of ε_{eff} = 45. The reconstructed image of head cross section utilizing the improved back-projection algorithm at (d) SNR = 20 dB, (e) SNR = 10 dB and (f) SNR = 5 dB.

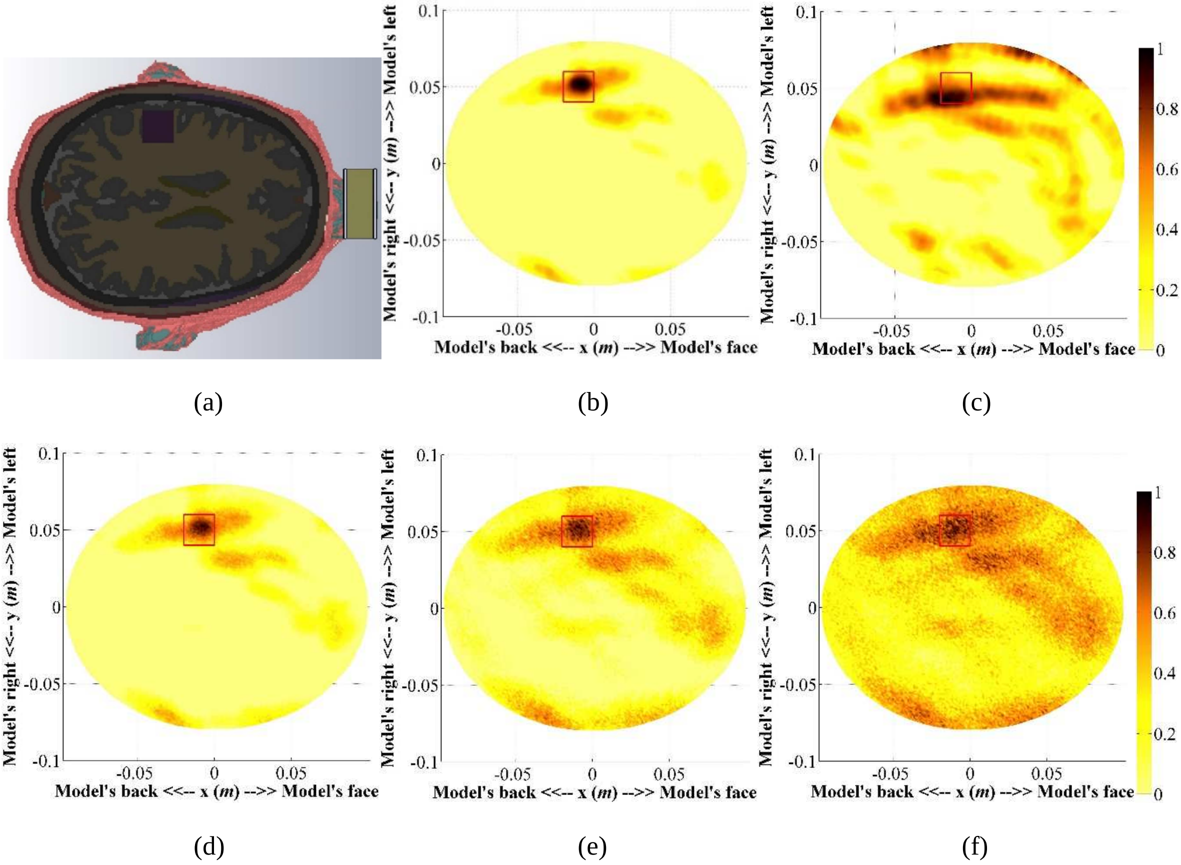

Supplementary Figure S25. (a) The representative scenario of realistic ICH affected human head at position 23 with the antenna placed in front of the head model. (b) The reconstructed image (SNR = 30 dB) of the head cross section using the proposed algorithm relying on the model of effective head permittivity. (c) Resulted image of the head cross section at the same situation and using the same dataset, but using the existing algorithm based on constant effective head permittivity of ε_{eff} = 45. The reconstructed image of head cross section utilizing the improved back-projection algorithm at (d) SNR = 20 dB, (e) SNR = 10 dB and (f) SNR = 5 dB.

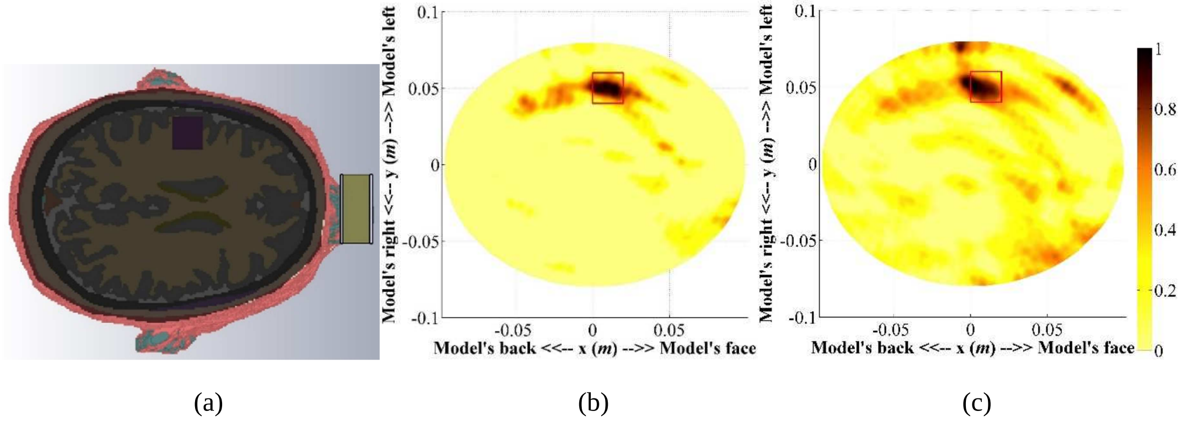

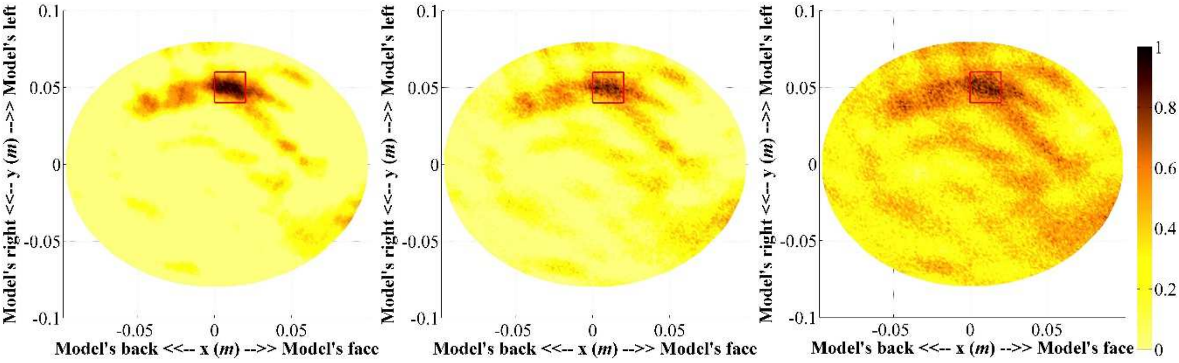

(d) (e) (f)

Supplementary Figure S26. (a) The representative scenario of realistic ICH affected human head at position 24 with the antenna placed in front of the head model. (b) The reconstructed image (SNR = 30 dB) of the head cross section using the proposed algorithm relying on the model of effective head permittivity. (c) Resulted image of the head cross section at the same situation and using the same dataset, but using the existing algorithm based on constant effective head permittivity of ε_{eff} = 45. The reconstructed image of head cross section utilizing the improved back-projection algorithm at (d) SNR = 20 dB, (e) SNR = 10 dB and (f) SNR = 5 dB.

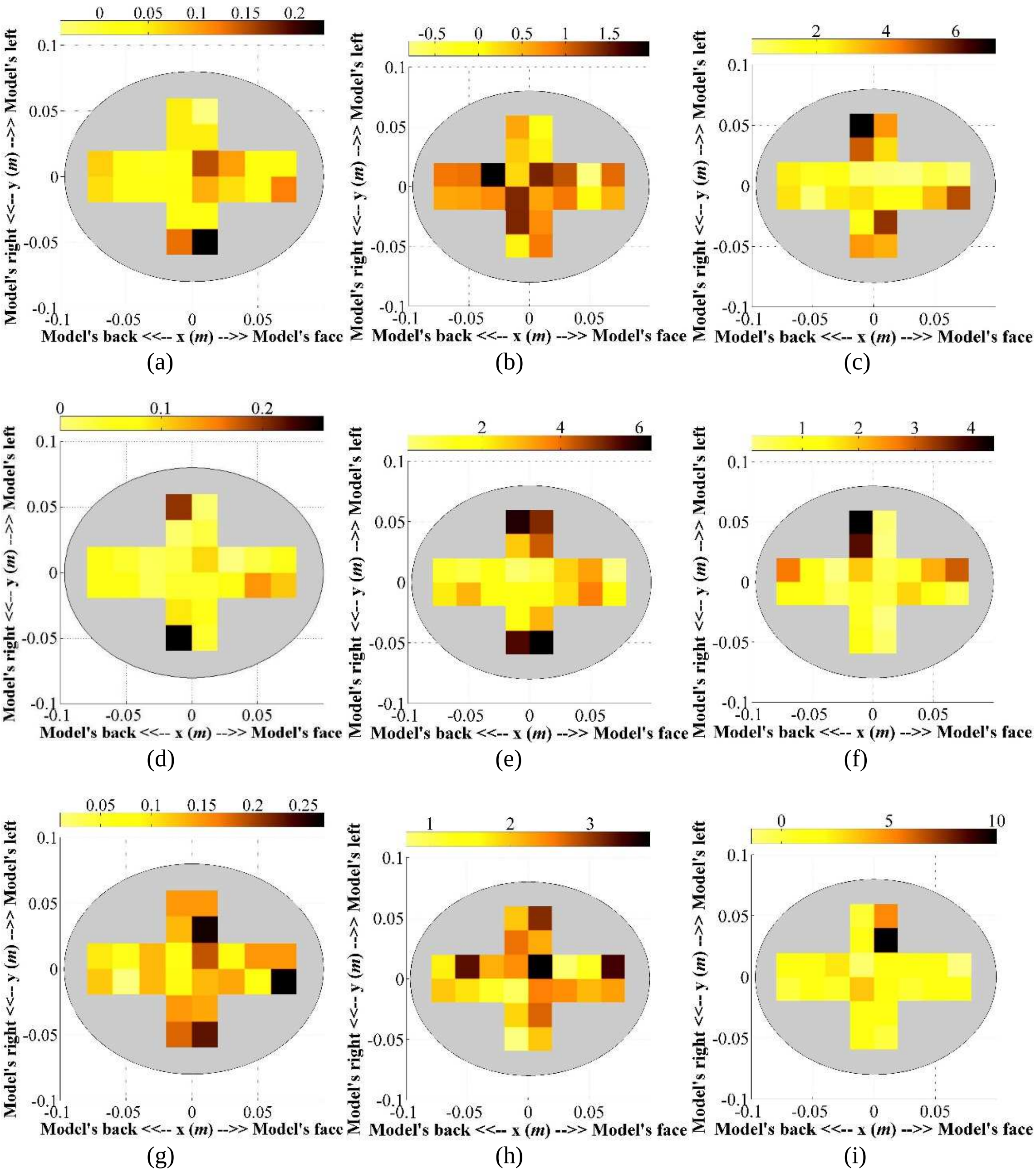

Supplementary Figure S27. (a-c) The differential map of the head model's cross section demonstrating the sensitivity of image reconstruction utilizing the proposed modified back-projection algorithm and when signal-to-noise ratio (SNR) reduces from 30 dB to 20 dB. (a) The map of differential signal to maximum clutter ratios, $\gamma_{diff1} = \gamma_{30dB} - \gamma_{20dB}$. (b) The differential map of average signal to clutter ratio functions, $Q_{diff1} = Q_{30dB} - Q_{20dB}$. (c) Difference map of the distances between true central location of the targets, $\delta_{diff1} = \delta_{20dB} - \delta_{30dB}$. (d-f) The differential map of the head model's cross section demonstrating the sensitivity of image reconstruction utilizing the proposed modified back-projection algorithm and when signal-to-noise ratio (SNR) reduces from 20 dB to 10 dB. (d) The map of differential signal to maximum clutter ratios, $\gamma_{diff2} = \gamma_{20dB} - \gamma_{10dB}$. (e) The differential map of average signal to clutter ratio functions, $Q_{diff2} = Q_{20dB} - Q_{10dB}$. (f) Difference map of the distances between true central location of the targets, $\delta_{diff2} = \delta_{10dB} - \delta_{20dB}$. (g-i) The differential map of the head model's cross section demonstrating the sensitivity of image reconstruction utilizing the proposed modified back-projection algorithm and when signal-to-noise ratio (SNR) reduces from 10 dB to 5 dB. (g) The map of differential signal to maximum clutter ratios, $\gamma_{diff3} = \gamma_{10dB} - \gamma_{5dB}$. (h) The differential map of average signal to clutter ratio functions, $Q_{diff3} = Q_{10dB} - Q_{5dB}$. (i) Difference map of the distances between true central location of the targets, $\delta_{diff3} = \delta_{5dB} - \delta_{10dB}$.
